# Stepwise surgical management of rectal foreign bodies: a multicenter operative case series and systematic review

**DOI:** 10.3389/fsurg.2026.1933279

**Published:** 2026-10-08

**Authors:** In Kyu Park, Kyung Uk Jung, Songsoo Yang, Sung Ryol Lee, Joosung Park, Hyung Ook Kim, Hungdai Kim, Jaemin Jo, Joon Young Choi, Sung Yoon Jang, Hongki Gwak, Weon Young Chang, Ho-kyung Chun, Donghyoun Lee

**Affiliations:** 1 Department of Surgery, Ulsan University Hospital, University of Ulsan College of Medicine, Ulsan, Republic of Korea; 2 Department of Surgery, Kangbuk Samsung Hospital, Sungkyunkwan University School of Medicine, Seoul, Republic of Korea; 3 Department of Internal Medicine, Jeju National University Hospital, Jeju National University School of Medicine, Jeju, Republic of Korea; 4 Department of Surgery, Jeju National University Hospital, Jeju National University School of Medicine, Jeju, Republic of Korea

**Keywords:** colorectal surgery, enterotomy, laparoscopy, milking, rectal foreign body, systematic review, transanal extraction, transanal minimally invasive surgery

## Abstract

**Background:**

Most rectal foreign bodies (RFBs) are removed non-operatively; operative management after failed transanal extraction remains poorly standardized. We describe a multicenter series of surgically managed RFBs and a systematic review of case series to quantify how often abdominal access avoids opening the bowel.

**Methods:**

All patients who underwent operating-room extraction of an RFB at three institutions over 5 years (2019–2023) were reviewed; bedside and endoscopy-suite removals were not captured, so the cohort represents surgically managed RFBs. The review (PubMed/MEDLINE, Embase, Scopus, Web of Science; Preferred Reporting Items for Systematic reviews and Meta-Analyses 2020; retrospective registration) included case series of five or more patients in any language, with extracted values checked against primary reports wherever available and source-level qualifications documented explicitly; all included series were appraised item by item with the Joanna Briggs Institute Critical Appraisal Checklist for Case Series.

**Results:**

In total, 22 patients (19 men; median age 49 years) were included, all self-inserted. Fourteen were managed transanally or by transanal minimally invasive surgery (TAMIS) (transanal 12, TAMIS 2) and eight by an abdominal approach (five open, two hand-assisted laparoscopic, and one laparoscopic); two minimally invasive attempts were converted. Two proximal objects were delivered per anum by milking without a deliberate bowel incision. Of the other six abdominal cases, three underwent resection, two required deliberate extraction enterotomies that were closed primarily, and one pre-existing contained perforation was managed by lavage and primary repair without diversion; a stoma was formed in three, all with perforation. There were no deaths. Abdominal cases more often involved objects above the rectosigmoid junction (7/8 vs. 2/14) and perforation (4/8 vs. 0/14), but not larger objects; all comparisons were exploratory and unadjusted. The review included 60 series from 26 countries (1976–2026). Among the 42 series that explicitly distinguished milking from bowel opening, 64 of 181 abdominal procedures were completed by milking without an enterotomy. Post-extraction endoscopy was performed in 0%–100% of patients across series.

**Conclusions:**

A stepwise, organ-preserving strategy—transanal or TAMIS extraction, then laparoscopic or hand-assisted milking, and then enterotomy, with resection and stoma reserved for perforation with severe injury or contamination—was executable in this cohort. In the published series that record the distinction, abdominal access did not entail opening the bowel in approximately one-third of procedures.

## 1 Introduction

Retained rectal foreign bodies (RFBs) are an uncommon but persistent surgical emergency. National administrative data from England recorded approximately 3,500 hospital episodes of manual removal of a rectal foreign body over 9 years—roughly one episode per day—at an estimated cost exceeding £3 million over a decade (1). Emergency-department surveillance data from the United States show an incidence rising from 1.2 to 1.9 per 100,000 between 2012 and 2021 (2), and single-institution series report a comparable trend (3, 4). Reported cohorts consistently show a marked male predominance and a bimodal age distribution, and presentation is frequently delayed by embarrassment, with patients often attempting self-extraction before seeking care (2, 3, 5, 6).

Although the condition has been described in the surgical literature for more than a century, management remains variably standardized. A stepwise principle—transanal extraction whenever possible, abdominal intervention as a last resort—was articulated as early as 1977 (7) and has been restated in single-center series and consensus documents across five decades (8–19). Yet practice varies widely between institutions in almost every dimension for which comparison is possible, including whether extraction is attempted at the bedside at all—some regard it as first-line (1, 20), while others explicitly advise against it (21, 22)—the use of post-extraction endoscopy (reported at rates from 0% to 100% across series), and the threshold for both abdominal surgery and fecal diversion.

Three aspects of this literature deserve particular attention.

First, “laparotomy” is frequently treated as synonymous with bowel opening and fecal diversion, and the fear of a stoma shapes both patient behavior and surgical decision-making. In fact, across the series we reviewed, a substantial fraction of abdominal procedures consisted of manual milking of the object toward the anus for transanal delivery, without colotomy, and colotomy itself was in some series closed primarily without diversion (4, 8, 10, 15, 23–25). The literature is explicitly divided on this point: several groups hold that colotomy mandates a stoma (3, 26), while others state the opposite as directly (17, 27–29).

Second, functional outcomes—sphincter integrity and continence after extraction—are almost never reported, not necessarily because they were ignored, but because patients in this population characteristically decline follow-up. This has been documented as a numerical finding: one series reported that 24 of 32 patients never attended outpatient review (30), another that every male patient declined the recommended post-extraction assessment (12), and others describe the same pattern (26, 31, 32).

Third, values reported in this literature are not always traceable to their primary sources. Summary tables in influential reviews contain summation errors and, in some instances, categories that do not exist in the original reports (29, 33), and a widely cited analysis of predictors of operative intervention (11) has been misquoted in four subsequent publications in a consistent direction, namely, converting statistically non-significant predictors into positive ones.

Several systematic reviews have addressed rectal foreign bodies, but their applicability to contemporary surgical practice is limited by outdated search periods or by restricted populations and geographic scope. The earliest reviewed reports that were published up to January 2009 (5). A subsequent meta-analysis was based on a search completed in 2018 (6). A later review was confined to men over 60 years (34) and a review published in 2026, identified through citation searching, was restricted to South Asia and rested on searches completed in March 2023 (35). None combines a current search with global scope and a focus on operative management. Notably, the earliest of these identified 17 case series but included only three, excluding the remaining 14 on the grounds that data on inserted, retained objects could not be separated from mixed cohorts (5). On examination of the original full texts, that judgment holds for some of those reports but not for most: in the majority, the eligible subgroup can be recovered from case-level tables or explicit statements in the text.

We therefore report a three-center operative case series in which operative practice was retrospectively characterized as a common stepwise approach, namely, a multicenter case-level report in which laparoscopic or hand-assisted milking and transanal minimally invasive surgery (TAMIS) were performed within that approach rather than only recommended, together with a systematic review in which extracted data were checked against primary reports wherever available, with limited source-level qualifications documented explicitly, and without language restriction.

The aims of this study were as follows: (1) to describe the operative management and short-term functional outcomes of the case series; (2) to synthesize, from primary sources, the proportion of abdominal interventions that avoid bowel opening (milking) and the frequency of fecal diversion; and (3) to characterize the variability of post-extraction endoscopic assessment and of functional follow-up across the published literature.

## 2 Methods

### 2.1 Study design and reporting standards

This study comprises (a) a retrospective multicenter operative case series and (b) a systematic review of published series of retained rectal foreign bodies. The case series has been reported in line with the Preferred Reporting Of CasE Series in Surgery (PROCESS) 2025 guideline (36), and the systematic review in accordance with Preferred Reporting Items for Systematic reviews and Meta-Analyses (PRISMA) 2020 (37). The review protocol was registered retrospectively on the Open Science Framework after the original submission, in the course of this revision (registration DOI: 10.17605/OSF.IO/A2XES); the retrospective nature of the registration is stated here explicitly.

### 2.2 Case series

#### 2.2.1 Setting and patients

Between January 2019 and December 2023, consecutive adult patients who underwent operative management of a retained rectal foreign body at three institutions (Jeju National University Hospital; Ulsan University Hospital; Kangbuk Samsung Hospital) were identified. The denominator of this series is operative cases: patients whose foreign bodies were removed in the emergency department without anesthesia were not captured by the operative registries used for case identification. This is a structural feature of operative series that has been acknowledged since Crass et al. (1981) and was restated in 2023 as a limitation shared by the two largest North American operative datasets (2); its implications are addressed in the Limitations.

#### 2.2.2 Ethics

The study was approved by the Institutional Review Board of Jeju National University Hospital (JEJUNUH 2026-07-012). The Institutional Review Boards of Ulsan University Hospital (UUH 2026-08-012) and Kangbuk Samsung Hospital (KBSMC IRB 2026-08-031) determined the study to be exempt from full review, on the grounds that it was a retrospective analysis of existing de-identified clinical records. The requirement for written informed consent was waived at all three centers for the same reason. Identical wording is used in the Declarations and in the journal ethics statement.

#### 2.2.3 Management strategy

Operative practice at the three centers was retrospectively characterized from the operative records and followed a common stepwise sequence: (1) assessment for peritonitis, with immediate abdominal exploration if present; (2) attempted transanal extraction under appropriate anesthesia; (3) where transanal extraction failed, abdominal access by laparoscopy or hand-assisted laparoscopy (HALS) where feasible, with manual milking of the object distally for transanal delivery and without a deliberate extraction enterotomy; (4) extraction enterotomy only where milking failed; and (5) resection or diversion reserved for perforation with severe bowel-wall injury, peritoneal contamination, or non-viable bowel. TAMIS was employed for objects lying low in the rectum that were palpable but beyond the reach of a finger or of forceps under direct vision, where a sealed platform with insufflation and instrument triangulation allowed extraction without abdominal access. Post-extraction rigid or flexible endoscopy was performed at the discretion of the attending surgeon rather than by protocol; digital assessment of sphincter tone was undertaken on postoperative days 1 and 3. The resulting variation in endoscopic assessment (performed in 10 of 22 patients) is reported as observed and is considered in the Discussion alongside the wide variation in this practice across the published literature.

Procedures were performed by attending surgeons at the three participating centers. The choice of operative approach was made by the operating surgeon according to the clinical circumstances, the characteristics and location of the object, and the presence of perforation or contamination. There was no common written protocol across the three centers; the stepwise sequence described here was reconstructed retrospectively from the operative records and represents a shared pattern of practice rather than a prospectively mandated algorithm.

#### 2.2.4 Variables, definitions, and outcomes

Data were abstracted from electronic medical records, operative notes, imaging reports, and outpatient records by one investigator at each center using a common data form, and independently checked against the source records by a second investigator; discrepancies were resolved by joint review of the primary record.

The following were recorded: age; sex; time from insertion to presentation; object type, longest dimension and maximum diameter; object material, categorized *post hoc* by the investigators as organic/deformable, rigid/brittle, rigid/non-brittle, elastomeric, or rigid plastic/wood on the basis of operative records, imaging descriptions, and inspection of the retrieved object where available (inter-rater reliability was not assessed, and the classification was not used for statistical testing); imaging performed and findings; object location, dichotomized at the rectosigmoid junction on digital examination and imaging; anesthetic technique; initial and completed operative approach; whether the bowel was opened; fecal diversion; complications; length of stay; and follow-up.

##### 2.2.4.1 Perforation

Perforation was defined as free intraperitoneal air on imaging or intraoperative confirmation of a full-thickness defect. All four perforations in this series were identified on computed tomography (CT) before the operation and confirmed intraoperatively, including the contained perforation with viable bowel that was managed by lavage and primary repair without diversion; free intraperitoneal air was therefore present in all four patients with perforation.

##### 2.2.4.2 Approach

Approach was classified by the completed procedure as follows: transanal (manual or instrumental), TAMIS, or abdominal (open, hand-assisted laparoscopic, or laparoscopic). Minimally invasive attempts that were converted were classified by the completed approach and annotated. Opening the bowel for extraction denotes a deliberate extraction enterotomy or resection; repair of a pre-existing perforation was recorded separately. Delivery of the object to the anus by transmural manipulation without a deliberate bowel incision (milking) is not counted as opening the bowel for extraction. Fecal diversion denotes a stoma formed at the index operation.

Complications were graded using the Clavien–Dindo classification and comprised postoperative adverse events; the index extraction, enterotomy, resection, or planned diversion was not itself counted as a complication. Post-extraction endoscopic assessment was performed at the discretion of the attending surgeon rather than by protocol, and its performance and findings are reported as observed. Sphincter assessment consisted of digital examination of resting and squeeze tone on postoperative days 1 and 3 and at outpatient review; anorectal manometry, endoanal ultrasound, and validated continence scores were not used. Continence was recorded from history and digital examination. Minimum follow-up was 2 months.

Circumstances of insertion were recorded neutrally as self-insertion; sexual orientation was neither recorded nor inferred. There were no missing data for the primary variables; where an item was unavailable (object dimensions in one patient) the denominator is stated.

Continuous variables are reported as median (range) and categorical variables as counts. Abdominal and transanal/TAMIS groups were compared using the Mann–Whitney U test and Fisher's exact test (two-sided) in Stata 17 (StataCorp, College Station, TX, USA). All comparisons are exploratory and unadjusted for multiplicity; because object location and perforation contributed to the selection of the operative approach, the associations reported characterize decision-making rather than independent prediction. No multivariable analysis was performed.

### 2.3 Systematic review

#### 2.3.1 Eligibility criteria

We included case series of five or more patients reporting the management of foreign bodies retained in the rectum after insertion per anum, in any language and any publication year, provided that the route of extraction (transanal vs. abdominal) could be determined. We excluded single case reports and series of fewer than five patients, cohorts restricted to ingested foreign bodies or to penetrating anorectal trauma, administrative or registry analyses in which extraction route or operative outcomes could not be derived (1, 2, 38, 39), narrative and systematic reviews without original patient data, and forensic or autopsy series.

Mixed cohorts (insertion together with ingestion, iatrogenic migration, or trauma) were handled in two ways. Where case-level data or an explicit statement allowed the transanally inserted subgroup to be identified, that subgroup alone was extracted and is the value entered in Supplementary Table S6 (10, 12, 27, 40, 41). Khan (2021) is included in this separable group because the eligible 15-patient subgroup could be extracted from the 22-patient parent cohort. Where the subgroup could not be separated, the study was retained only if the extraction route was reported for the whole cohort and the report identified the ineligible cases individually or stated that they were a minority of the cohort; for these studies, the whole-cohort value is entered and the row is footnoted in Supplementary Table S6. Nine included studies fall into this non-separable group. This review-level classification of subgroup separability was recorded independently of the Joanna Briggs Institute (JBI) appraisal; in particular, JBI item 1 was judged solely on whether the original report stated clear criteria for inclusion in its own case series. A sensitivity analysis excluding the nine non-separable cohorts is reported in Results and in Supplementary Table S4. Cohorts including minors were not excluded on that basis alone; the age range of each series is given in Supplementary Table S6 and series containing patients under 18 years old are footnoted.

Where two or more reports described the same cohort, the report with the broader denominator was designated the primary record and the others were treated as linked publications and not counted separately. Four such pairs were identified (30, 40, 42, 43); one further pair from the same institution and era was found not to overlap and both reports were retained (44).

Primary endpoints for extraction were as follows: cohort size; abdominal access; among abdominal procedures, milking (transanal delivery without bowel opening) vs. colotomy vs. resection; fecal diversion; and mortality. Secondary items were post-extraction endoscopic assessment (performance and yield), sphincter injury and continence outcomes, anesthesia practice, and the presence of an institutional algorithm.

#### 2.3.2 Search strategy

The search strategy submitted with the original manuscript contained a structural flaw identified during this revision: an AND-clause of operative terms functioned as a filter, excluding eligible series whose abstracts did not contain those terms (e.g., Caliskan et al. 2011 and Brungardt et al. 2021). The strategy was therefore rebuilt: the operative AND-clause was removed, and MeSH and Emtree controlled vocabulary were added. The revised search was run on 5 August 2026 in PubMed/MEDLINE (1,465 records), Embase (2,312), Scopus (1,857), and Web of Science Core Collection (384)—6,018 records in total—with no restriction by language, publication date, or intervention. All 18 series included in the original submission were retrieved by the revised strategy, supporting its sensitivity. After removal of 2,713 duplicates, 3,305 records were screened. Full search strings for each database are provided in Supplementary Table S5.

Forward citation searching of the two prior systematic reviews addressing the condition as a whole (5, 6) yielded 105 citing records; none met the eligibility criteria. Because this citation-screening activity is at a different level from the 38 report-level records identified through other methods, it is reported descriptively and is not added to the PRISMA report-level counts.

Backward citation searching was undertaken for all included series with 30 or more patients, all included series published from 2010 onward, and six narrative or systematic reviews of the topic (5, 6, 13, 17, 29, 35). This route identified additional candidate reports, but final PRISMA pathway assignment was based on presence in the four original database exports rather than on the order in which a report came to notice. Reports absent from all four exports and identified through citation or targeted journal searching were assigned to the other-methods pathway; full-text exclusions are itemized by reason in Supplementary Table S2.

Because several eligible reports were identified outside the four database search outputs, three targeted supplementary searches were performed: a regional bibliographic database using five search terms in English and Chinese, which returned no additional eligible series; the online archives of two national colorectal journals; and a Russian-language bibliographic database, access to which was not available from our location. Reports identified by citation searching and by these targeted searches entered the retrieval stage alongside reports advancing from the database screen. Pathway assignment was determined by presence in the four original database exports rather than by the order in which a report came to notice: reports present in any export were counted in the database pathway even where citation searching had drawn attention to them first.

#### 2.3.3 Study selection

Titles, abstracts, and full texts were screened independently and in duplicate by two reviewers (DL and SRL); disagreements were resolved by discussion, and where consensus was not reached, a third reviewer (KUJ) adjudicated.

#### 2.3.4 Full-text retrieval

Because a substantial portion of the eligible literature predates electronic archiving or appeared in journals that have since ceased publication, full-text retrieval required measures beyond database access, including document supply from the library and archive of the Royal College of Surgeons of Edinburgh, which holds bound volumes of its discontinued journal and supplied two reports (45, 46); institutional document-delivery services; commercial document supply; open repositories; and direct contact with authors and colleagues holding the original articles.

Several titles relevant to this review begin their electronic archives after the year of the report sought, and the volumes of one national colorectal journal published before 2008 are neither indexed nor digitized. One holding library was able to supply only the abstract page of a 1983 report, which was subsequently obtained in full through a commercial document-delivery service; its study period (1978–1981) did not overlap that of the same group's earlier report (1970–1974), and the two were therefore retained as independent cohorts.

Fourteen of the included series were published in a language other than English, namely, Russian (eight) (18, 24, 25, 27, 43, 47–49), Turkish (three) (23, 50, 51), German (one) (33), Japanese (one) (52), and Spanish (one) (53). Access to Russian-language medical literature has become more difficult since 2022, when Clarivate suspended operations in Russia and ceased evaluating Russian journals for the Web of Science, and several major publishers suspended services in the region; older non-indexed titles are particularly affected.

Nine reports could not be obtained in full text despite these measures and are recorded as “reports not retrieved” in the PRISMA flow diagram (54–62). These references are cited solely to identify the unavailable reports; they were not included in the review, and no data from them, including abstract-only data, were used in the synthesis. Five of these are reports in a Turkish national colorectal journal whose volumes published before 2008 are neither indexed nor digitized; they could not be obtained despite direct enquiry to the publishing society or authors. One of them, Korkut et al. (1995), is described in its title as a retrospective series of five patients and would probably have been eligible.

#### 2.3.5 Data extraction and verification

Data were extracted from the primary report of each included study wherever available. The source used for each report is recorded in the review dataset, and any qualification involving a subsequent report or author clarification is identified explicitly in Supplementary Tables S3, S6. Values quoted in prior reviews or in the discussion sections of other papers were not used as a data source. This decision was not precautionary but empirical: on comparison with the primary reports, summary tables in two influential reviews were found to contain summation errors, to combine mutually exclusive categories, and in two instances, to present a division of the cohort that does not exist in the original article (29, 33). The propagation of misquotations of Lake et al. (2004) in four subsequent publications is described in the Discussion.

Prompted by the reviewers’ comments, every value carried from the original submission was re-verified against the primary full text, with one exception: for Ayantunde and Oke (2006), the size of the eligible rectal subgroup (*n* = 16) was confirmed from the authors’ subsequent report because the subgroup table in the 2006 full text was not available to us. During the final archival closure sweep, Trenin (2005) was added as a non-separable mixed cohort: its whole-cohort denominator and abdominal-procedure count were retained with the mixed-cohort flag, while classification of three of the four abdominal procedures as delivery to the anus without bowel opening was supported by clarification from the corresponding author of a later Russian series, as disclosed in Supplementary Table S6. Entries that were inaccurate or uncertain were corrected; the corrections are itemized in Supplementary Table S3, including revision of the Barone 1976 cohort from 28 to 23 (five perforation cases in which the foreign body had already passed before presentation did not meet the inclusion criterion of a retained foreign body).

Non-English full texts were translated with the aid of machine translation and extracted with the numeric tables cross-checked against the original-language text; items that could not be resolved with confidence were recorded as unclear rather than inferred.

Where a study reported multiple denominators (patients, presentations, foreign bodies), the denominator used is stated in Supplementary Table S6, and discrepancies internal to a study (abstract vs. text vs. tables) are annotated with the value adopted and the reason.

#### 2.3.6 Critical appraisal

The JBI Critical Appraisal Checklist for Case Series (10 items) was used for methodological appraisal (63). The Joanna Briggs Institute is revising its quantitative appraisal tools in sequence (randomized trials, quasi-experimental studies, cohort studies, and analytical cross-sectional studies), but the case series checklist has not been superseded, and the 2020 tool remains the current version. All 60 included studies were appraised independently by two reviewers (DL and SRL), who assessed each study in parallel; disagreements were referred to a third reviewer (KUJ) for adjudication.

The composite grading used in the original submission—author-defined count thresholds yielding “low” and “moderate” labels—was abandoned. The developers of the tool advise against cut-off scores, on the grounds that the items are not of equal weight and that tallying “yes” responses does not indicate the specific problems of a study, and they recommend presenting the appraisal for all questions in a table rather than as a score (63). We therefore report the item-level judgments (yes/no/unclear/not applicable) in Supplementary Table S7, with the full item wording in the table footnote. For item 1, judgments were based solely on whether the original report stated clear criteria for inclusion in its own case series; mixed-cohort separability under the eligibility criteria of this review was assessed separately and did not determine the item-1 judgment. For item 10, “not applicable” was used only when the report did not undertake a statistical analysis whose appropriateness could be appraised; otherwise, the reported analysis was judged yes, no, or unclear. “Not applicable” judgments were excluded from any denominator, and no summary score was calculated.

#### 2.3.7 Synthesis

Given the heterogeneity of designs, eras, and denominators, results were synthesized descriptively. We tabulated, per study, the following: cohort size and eligible subgroup; abdominal access; milking, colotomy, and resection within abdominal procedures; diversion; mortality; post-extraction endoscopy (performance and yield); and functional follow-up. No meta-analytic pooling was performed. Where era-level patterns are described (e.g., the proportion of abdominal interventions over time), they are presented as descriptive observations anchored to the per-study values in Supplementary Table S6, not as pooled estimates.

## 3 Results

### 3.1 Case series

In total, 22 patients were treated over 5 years (Table 1). The median age was 49 years (25–74); 19 (86.4%) were male, and all objects were self-inserted. The median time to presentation was 8 h (0.5–36). Objects were diverse, including bottles and glasses, aerosol and cosmetic containers, sexual devices, vegetables, and wooden and metallic handles, with a median longest dimension of 17 cm (5–30); nine (40.9%) lay above the rectosigmoid junction. Seven patients underwent plain radiography alone and 15 also underwent CT, which was used selectively for suspected high-lying objects or possible perforation; free air was present in four (18.2%), corresponding exactly to the four patients whose perforations were confirmed at operation.

**Table 1:** Baseline, object, imaging, and treatment characteristics (*N* = 22).

Characteristic	Value
Age, years, median (range)	49 (25–74)
Sex, *n* (%)	
Male	19 (86.4)
Female	3 (13.6)
Self-insertion, *n* (%)	22 (100)
Time to presentation, hours, median (range)	8 (0.5–36)
Object length (longest dimension), cm, median (range), *n* = 21	17 (5–30)
Object material category, *n* (%)	
Organic/deformable	2 (9.1)
Rigid/brittle	3 (13.6)
Rigid/non-brittle	8 (36.4)
Elastomeric	3 (13.6)
Rigid plastic/wood	6 (27.3)
Object location, *n* (%)	
Above rectosigmoid junction	9 (40.9)
At or below rectosigmoid junction	13 (59.1)
Imaging, *n* (%)	
Plain radiograph only	7 (31.8)
Radiograph and CT	15 (68.2)
Free air	4 (18.2)
Perforation confirmed at operation, *n* (%)	4 (18.2)
Completed approach, *n* (%)	
Transanal, manual or instrumental	12 (54.5)
TAMIS-assisted	2 (9.1)
*Transanal or TAMIS, subtotal*	*14* (*63.6)*
Abdominal (open 5; HALS 2; laparoscopic 1)	8 (36.4)
Converted from a minimally invasive attempt	2 (9.1)
Procedure performed within abdominal approach, *n* (%)	
Milking to the anus without deliberate bowel incision	2 (9.1)
Deliberate bowel opening for extraction	5 (22.7)
Extraction enterotomy with primary closure	2 (9.1)
Resection	3 (13.6)
Repair of a pre-existing contained perforation without extraction enterotomy	1 (4.5)
Stoma formation, *n* (%)	3 (13.6)
Post-extraction endoscopic assessment, *n* (%)	10 (45.5)
Mucosal laceration identified, n/n examined	6/10
Transient incontinence to liquid stool, *n* (%)	3 (13.6)
Clavien–Dindo grade, *n* (%)	
0	12 (54.5)
I	5 (22.7)
II	3 (13.6)
IIIb	2 (9.1)
In-hospital mortality, *n* (%)	0 (0)
Length of stay, days, median (range)	2 (1–16)
Follow-up, months, minimum	2

Approaches are classified by the completed (final) approach; two abdominal cases began as a minimally invasive attempt that was converted. Free air on imaging corresponded exactly to the four perforations confirmed at operation. Milking denotes delivery of the object to the anus by transmural manipulation without a deliberate bowel incision and is not counted as opening the bowel for extraction. Deliberate bowel opening for extraction comprises two extraction enterotomies and three resections; repair of the pre-existing contained perforation is recorded separately. Post-extraction endoscopic assessment was performed at the discretion of the attending surgeon rather than by protocol. The three episodes of transient incontinence are included among the five Clavien–Dindo grade I events and had resolved within 2 months. Object material categories were assigned *post hoc* by the investigators; percentages may not sum to 100 because of rounding.

CT, computed tomography; HALS, hand-assisted laparoscopic surgery; TAMIS, transanal minimally invasive surgery.

By completed (final) approach, 14 patients (63.6%) had been managed transanally or with TAMIS—manual or instrumental transanal extraction in 12 and TAMIS-assisted extraction in 2—and 8 (36.4%) by an abdominal approach, including open in 5, HALS in 2, and laparoscopic in 1 (Table 1; case-level detail in Supplementary Table S1). Two of the abdominal cases began as a minimally invasive attempt that was converted (case 16, laparoscopic to HALS; case 19, laparoscopic to open).

Considering the actual procedure rather than the route, two of the eight abdominal cases were completed by laparoscopic or hand-assisted milking without a deliberate bowel incision or stoma (cases 14 and 16). Of the remaining six, three underwent resection with stoma, two required deliberate extraction enterotomies that were closed primarily, and one (case 19) had a pre-existing contained perforation managed by lavage and primary repair without diversion. The perforation in case 19 had been identified on preoperative CT and was confirmed intraoperatively. One of the two extraction-enterotomy cases was managed by HALS after transanal extraction failed because of the object's wedge shape. A stoma was formed in three patients (13.6%): two Hartmann procedures and one low anterior resection with diverting ileostomy. There were no deaths.

Post-extraction proctoscopy or flexible sigmoidoscopy was performed at the discretion of the attending surgeon in 10 of 22 patients (45.5%). Because endoscopy was discretionary and was not a prespecified route-comparison outcome, no approach-specific performance denominator is reported. Mucosal lacerations were identified in 6 of the 10 examined (60%); the submucosa was intact in every case, and no injury identified at post-extraction endoscopy altered management. Digital rectal examination was performed on postoperative days 1 and 3 in all patients; resting and squeeze tone were recorded as preserved, and no clinically apparent sphincter deficit was documented. Anorectal manometry and pelvic magnetic resonance imaging were not performed. Transient incontinence to liquid stool was reported by three patients (13.6%), all of whom had undergone manual transanal extraction; each was identified at the first outpatient visit, all were assessed by history and digital examination, and all had resolved within 2 months. These three episodes are included among the five Clavien–Dindo grade I events. All 22 patients had a minimum follow-up of 2 months.

Patients selected for an abdominal approach more often had a location above the rectosigmoid junction (7/8 vs. 2/14) and perforation (4/8 vs. 0/14); their median stay was longer (9 vs. 1.5 days) and they were older (median 57 vs. 40 years). Stoma formation occurred in 3 of 8 abdominal cases and in none of the 14 transanal or TAMIS cases (Table 2). Object dimensions did not differ between groups (available in 21 patients). Differences in time to presentation and in complication rate were not statistically significant. These comparisons are descriptive because location and perforation contributed to the operative approach selected.

**Table 2:** Abdominal vs. transanal or TAMIS management.

Variable	Abdominal (*n* = 8)	Transanal or TAMIS (*n* = 14)	*p*
Age, years, median (range)	57 (48–71)	40 (25–74)	0.015
Male, *n*	7/8	12/14	1.000
Location above the rectosigmoid junction, *n*	7/8	2/14	0.001
Perforation (free air), *n*	4/8	0/14	0.010
Time to presentation, hours, median (range)	15.5 (0.5–36)	5 (2–24)	0.060
Object maximum diameter, cm, median (range)^a^	5.0 (3.0–10.0)	4.0 (2.0–5.8)	0.331
Object length, cm, median (range)^a^	16 (5–28)	17.5 (5–30)	0.654
Complication of Clavien–Dindo grade I or higher, *n*	6/8	4/14	0.074
Stoma formation, *n*	3/8	0/14	0.036
Length of stay, days, median (range)	9 (2–16)	1.5 (1–6)	<0.001

Values are median (range) or *n*. *p* values are from the Mann–Whitney *U* test for continuous variables and Fisher's exact test for categorical variables. These comparisons are exploratory and descriptive: object location and perforation contributed to the choice of operative approach, so the corresponding differences are in part a consequence of the selection they describe rather than independent predictors of it. No adjustment for multiple comparisons was applied.

TAMIS, transanal minimally invasive surgery.

a Object dimensions were available for 21 patients (7 abdominal, 14 transanal or TAMIS); one abdominal case had unmeasured dimensions, and the *p*-values for object size are based on the available data.

### 3.2 Systematic review

#### 3.2.1 Study selection

The rebuilt database search retrieved 6,018 records (PubMed/MEDLINE 1,465; Embase 2,312; Scopus 1,857; Web of Science Core Collection 384). After removal of 2,713 duplicates, 3,305 database records were screened by title and abstract; 3,207 were excluded and 98 reports were sought for retrieval. Four could not be obtained, leaving 94 reports assessed in full text, of which 44 were excluded and 50 met the eligibility criteria. Separately, 38 reports identified outside the four database outputs—through backward citation searching and targeted journal searches—were sought for retrieval; five were not obtained, 33 were assessed in full text, 23 were excluded, and 10 were included. In total, 60 case series met the eligibility criteria (Figure 1; Supplementary Table S6).

**Figure 1:**
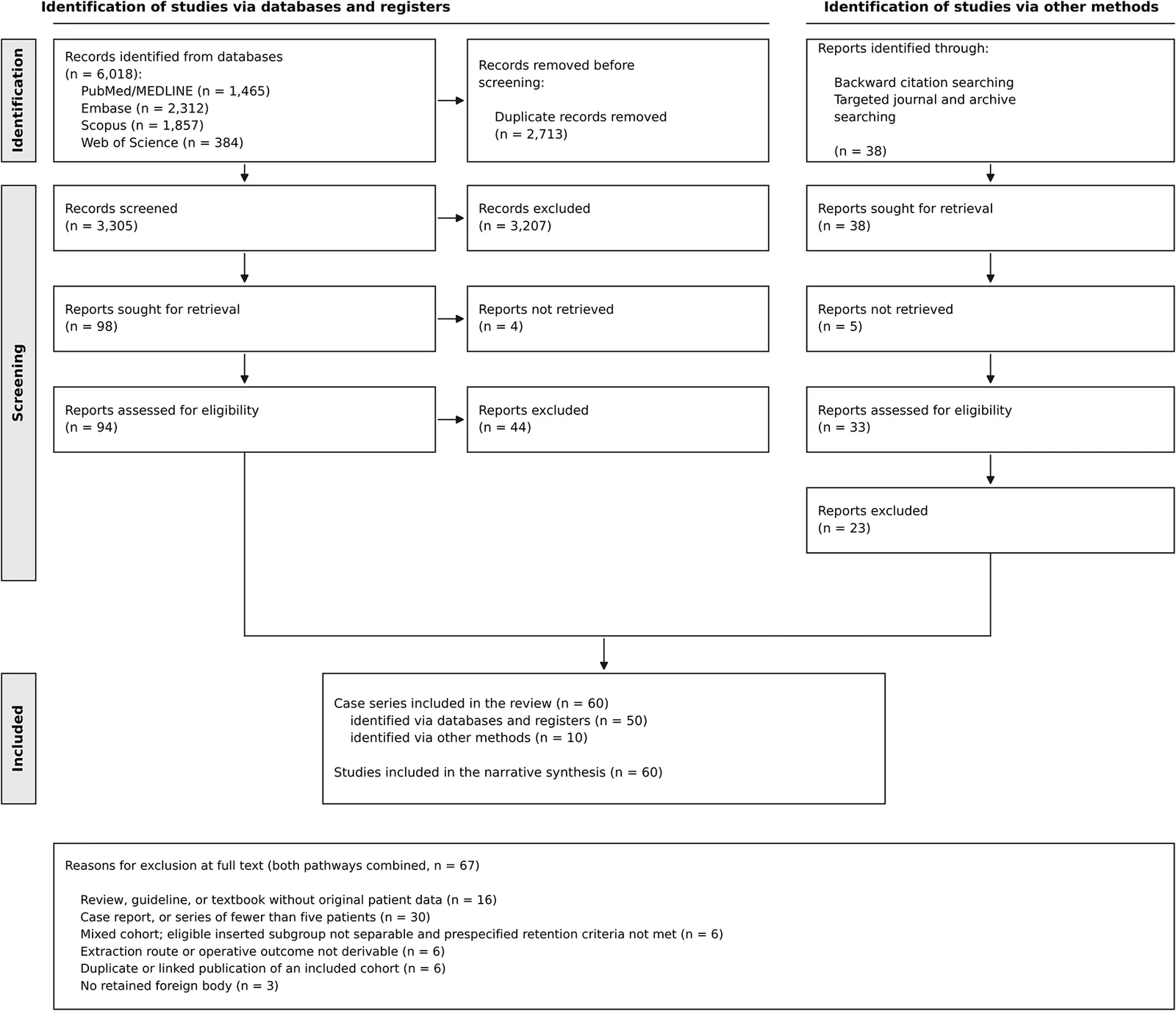
PRISMA 2020 flow diagram. The database searches were run on 5 August 2026 and citation searching on 6 August 2026, with no restriction by language, publication date, or intervention. Reports were assigned to a pathway based on their presence in the four original database exports as follows: a report present in any export remained in the database pathway even if it had first come to notice through citation searching, whereas a report absent from all four exports was assigned to the other-methods pathway. Forward citation searching of the two prior systematic reviews screened 105 citing records, none of which proved eligible.

The included series span 1976 to 2026 and 26 countries, and range in size from 5 to 648 patients. Fourteen series were published in five languages other than English (Russian, Turkish, German, Japanese, and Spanish). Additional included reports were identified outside the four database search outputs through citation searching and targeted journal searches.

#### 3.2.2 Abdominal access

Cohort denominators differed substantially between series and were of the following three types: whole-presentation cohorts including bedside extractions; operating-room or surgically referred cohorts; and administrative or registry datasets. Because of this, abdominal-procedure rates were not pooled as a summary estimate.

Across the 59 series in which both cohort size and the number of abdominal procedures could be determined, 428 of 2,867 patients underwent an abdominal procedure. This crude aggregate (14.9%) sums studies with different denominators and is not a summary estimate. The range was 0%–75.0%, and the distribution tracked cohort type rather than era alone: four series reported no abdominal procedure at all (44, 45, 51, 64), whereas the highest proportions were reported in an operating-room-only registry analysis (65) and in two series in which the majority of patients presented with established complications (16, 41). Our own 8 of 22 (36.4%) should be read within an operating-room-only denominator and is not comparable with whole-presentation cohorts.

#### 3.2.3 Milking vs. opening the bowel

In total, 42 series distinguished, within abdominal procedures, between manual milking of the object toward the anus for transanal delivery and opening the bowel. In these series, 64 of 181 abdominal procedures (35.4%) were completed by milking without an enterotomy. In five series, every abdominal procedure was completed in this way (8, 10, 23–25), and in two of these, the bowel was not opened, even in the presence of perforation, which was repaired without colotomy (24, 48).

Colotomy, where reported, could be closed primarily in selected cases; the published data do not permit a direct denominator for colotomy with vs. without diversion, because colotomy counts and stoma counts are drawn from different reporting subsets. Across the 42 series reporting colotomy counts, 85 colotomies were recorded among 254 abdominal procedures; this denominator differs from the 181 abdominal procedures underlying the milking proportion because the two reporting subsets are not identical. Practice on diversion after colotomy is explicitly divided in the primary literature: several groups state that colotomy is an absolute indication for a stoma (3, 26), while others state the opposite in equally direct terms (27, 28), and one series reported that both colotomies were closed primarily with no stoma formed in the cohort (15). At the other extreme, one series reported that every bowel opening ended in a stoma (66).

Milking has been recommended for far longer than it has been recorded as performed. It appears as an explicit step in guidance from 1977 onward (7, 9, 13, 16, 17, 19, 46), yet several series that recommend it in their discussion report no instance of its use in their own cohort (15, 33, 67). One series recorded the absence explicitly, stating that no attempt to milk the object distally was documented in any of the four cases in which the rectal wall was opened (21).

A stoma was reported in 151 patients across the 52 series reporting this outcome. In the great majority of series, diversion was confined to patients with perforation, peritoneal contamination, or non-viable bowel rather than to failed transanal extraction as such.

#### 3.2.4 Mortality

Deaths were reported in five series: 8 of 648 in a national administrative dataset (68), 3 of 30 in a single-center series in which one patient died before any extraction attempt (12), and one death each in three further series (3, 27, 43). In total, 54 series reported mortality, of which 49 reported none.

A compact summary of the principal systematic-review endpoints is provided in Table 3; study-level data are provided in Supplementary Table S6.

**Table 3:** Summary of principal systematic-review endpoints.

Endpoint	Reporting series	Result	Interpretation
Abdominal procedure	59	428/2,867 (14.9%)	Crude aggregate across heterogeneous cohort definitions; not a pooled estimate
Milking without bowel opening	42	64/181 (35.4%)	Restricted to series that explicitly distinguished milking from bowel opening
Colotomy	42	85/254	Reporting subset differs from the milking subset
Stoma formation	52	151 patients	No common patient denominator across reporting series
Mortality	54	14 deaths in 5 series; 49 series reported zero deaths	No values were imputed for non-reporting series

Abdominal and milking percentages are descriptive crude aggregates, not meta-analytic pooled estimates. Full study-level data, source qualifications, and row-specific notes are provided in Supplementary Table S6.

#### 3.2.5 Post-extraction endoscopic assessment

Practice varied across the full possible range. Endoscopic assessment after extraction was performed in every patient in several series (7, 8, 10, 14, 23, 69, 70) and in none in others (42). Intermediate rates included 79% (33), 76% (64), 45.5% (present series), 22.7% (15), and 7% (71).

Where the diagnostic yield was also reported, injury was identified in a substantial proportion of those examined, as follows: 100% (31, 44, 72), 62.5% (22), 60% (present series), 57% (67), 35% (8), 33% (14), 28.6% (23), 26.7% (70), and 20% (15).

Recommendation and practice diverged within individual reports. One series concluded that post-extraction endoscopy should be part of a standardized pathway while reporting its own performance rate as 22.7% (15); another described flexible sigmoidoscopy as standard practice after removal while reporting that it was not performed in any patient, and stated the reason—endoscopy services in that trust were fully committed (42). A third group reached the opposite conclusion from a 7% performance rate, arguing that routine examination is not necessary (71). Consensus guidance reflects this division, offering only a weak recommendation and noting that the practice is debated (17).

#### 3.2.6 Sphincter injury and continence

Sphincter injury was recorded in several series but its functional consequence was almost never followed. Two internal sphincter disruptions were reported without any statement of their management or outcome (20); an external sphincter laceration was repaired primarily with no follow-up after discharge (45); an internal sphincter tear was recorded without functional data (73).

The reason is documented in the primary literature and is not simply inattention. One group advised transrectal ultrasound and intervention for incontinence or stenosis after discharge and reported that every male patient declined (12); another reported that 24 of 32 patients never attended outpatient review (30); others describe the same pattern, in one case attributing it to the stigma attached to the presentation (26, 31, 32).

A minority of series report continence outcomes; among the few patients with documented follow-up, outcomes were generally favorable. The longest follow-up is a telephone interview at a median of 63 months with complete response, in which every patient was continent to gas, liquid, and solid stool (10). Others report no incontinence following anal dilatation (46, 69), no continence disturbance after stoma reversal (53), normal continence at 1 year (74), and preserved continence at discharge after primary repair of sphincter disruption (20). Our own finding, i.e., transient liquid-stool incontinence in 3 of 22 patients, all resolving within 2 months, is consistent with this pattern.

Sphincterotomy remains contested. Lateral or posterior sphincterotomy is described as an aid to extraction of large objects in several reports (7, 16, 23, 26, 46, 75) and as unavoidable for very large objects in one (49), while others advise explicitly against it on the grounds that the sphincter mechanism is at least partly destroyed (6, 27). Only one series we identified reports both the frequency with which it was performed and its functional consequences: among 11 patients in whom transanal extraction was combined with posterior sphincterotomy, 5 reported anal pain on defecation, 4 reported incontinence to flatus, and 2 developed suppuration of the sphincterotomy wound, with follow-up limited to the 4–7 days to discharge (43).

#### 3.2.7 Methodological quality

Item-level appraisals are presented in Supplementary Table S7; no composite score was calculated. Across the 60 included series, clear reporting was most consistent for demographics (item 6: 60/60 yes), identification methods (item 3: 53/60 yes), measurement of the condition (item 2: 52/60 yes), and presenting site/clinic information (item 9: 55/60 yes). Consecutive inclusion was unclear in 36 of 60 studies, and complete inclusion was unclear in 41 of 60. Item 10 was not applicable in 49 studies because no inferential statistical analysis was undertaken; among the 11 studies with an analysis that could be appraised, eight were judged appropriate, two unclear, and one inappropriate because a proportion was compared with a t-test after the authors had stated that no detailed statistical analysis was performed (76). Mixed-cohort separability is a review-level eligibility variable rather than a JBI item. Excluding the nine non-separable mixed cohorts changes the aggregate proportion of abdominal procedures from 428 of 2,867 (14.9%) to 303 of 1,908 (15.9%) (Supplementary Table S4). Khan (2021) was not excluded in this analysis because its eligible 15-patient subgroup was separable from the 22-patient parent cohort.

Two patterns are worth noting because they bear on how the checklist should be read.

Across all 60 appraisals, complete inclusion (item 5) was judged “no” in eight studies, in every case because the authors disclosed the loss—nine chart records not found in one study, eight records with insufficient documentation in another, two patients who left against medical advice in a third, and, in five further studies, the exclusion of records with incomplete data. Studies that made no statement sufficient to establish complete inclusion were judged “unclear.” This pattern should be interpreted cautiously, because explicit reporting of participant loss resulted in a “no” judgment, whereas absence of information resulted in “unclear.”

Across all 60 appraisals, clear reporting of clinical information (item 7) was judged “no” in eight studies for two different reasons: in four, the data source itself does not contain object characteristics or presentation details (30, 65, 68, 73), and the authors of two of these state so explicitly; in the other four, case-level information was simply not reported although it would have been available (19, 32, 43, 77).

##### 3.2.7.1 JBI item 1 was evaluated independently of review eligibility

JBI item 1 reflects whether the original report stated clear criteria for inclusion in its own case series, not whether an inserted-RFB subgroup was separable for this review. The non-separable mixed-cohort classification used in the sensitivity analysis is therefore reported separately and does not determine the item-1 judgment.

## 4 Discussion

### 4.1 Principal findings

In this multicenter series of 22 surgically managed rectal foreign bodies, the majority of patients were treated with organ-preserving techniques: 14 of 22 were removed transanally or by TAMIS, and among the eight managed by an abdominal approach, two proximal objects were delivered from the anus by laparoscopic or hand-assisted milking without a deliberate bowel incision. Among the other six abdominal cases, three underwent resection, two underwent extraction enterotomy with primary closure, and one pre-existing contained perforation with viable bowel was managed by lavage and primary repair without diversion; a stoma was formed in the three resection cases. There were no deaths.

Read alongside 60 published series from 26 countries, these findings support the following three propositions: that abdominal access and opening the bowel are separable decisions; that the reported range of abdominal intervention reflects cohort composition rather than era; and that the near-absence of functional outcomes in this literature has a documented structural cause rather than being an oversight.

The stepwise principle itself is not new. Transanal extraction first, abdominal access as a last resort, and opening the bowel only when milking fails was set out in 1977 (7) and restated across five decades (8, 9, 13, 16, 17, 19, 46). Nor is TAMIS for foreign-body retrieval new; the transanal minimally invasive platform was described in 2010 for local excision of rectal neoplasia (78), and its use for foreign-body extraction has been reported since 2012 and repeatedly thereafter (27, 79–84). Adjunctive maneuvers occupying the same intermediate space have been described, including endoscopic balloon dilatation of a spastic segment (85) and combined endoscopic and surgical retrieval (86). The same logic, i.e., transanal and abdominal minimally invasive access as complementary components of a single operation rather than as alternatives, has recently been applied outside foreign-body retrieval, in a technique combining a transanal platform for local excision with laparoscopic abdominal access within one procedure (87). What is scarce in the literature is not the recommendation but its execution: in the series we reviewed, laparoscopic or hand-assisted milking was recommended far more often than it was recorded as performed. The common stepwise approach retrospectively characterized across the three centers is summarized in Figure 2. The contribution of the present work is therefore not the sequence but its case-level execution—a multicenter report in which laparoscopic and hand-assisted milking and TAMIS were actually carried out within that common stepwise approach, together with a synthesis quantifying how often the intermediate steps have in fact been used elsewhere.

**Figure 2:**
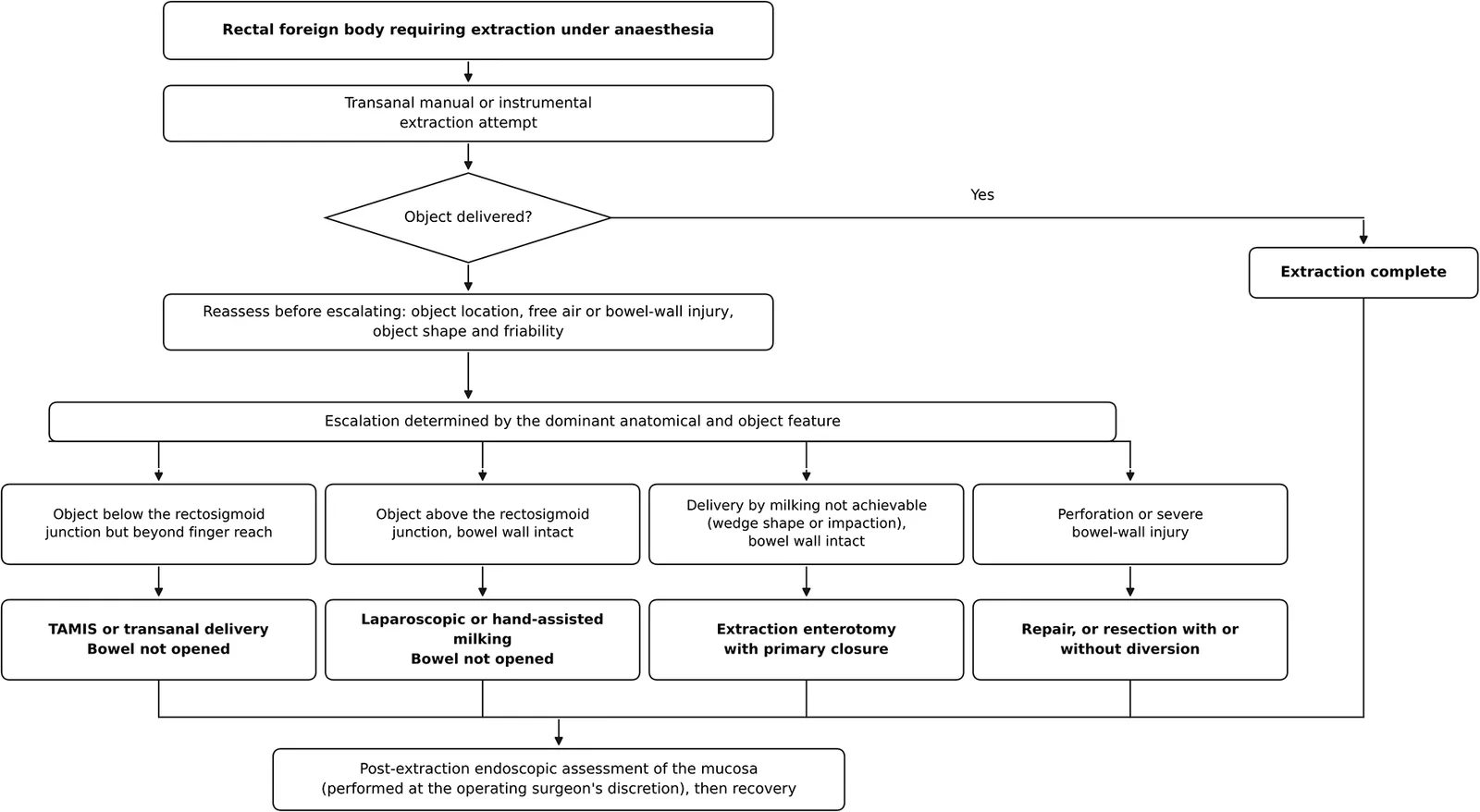
A stepwise, organ-preserving approach to a retained rectal foreign body requiring extraction in the operating room, as practiced across the three participating centers. Escalation proceeds downward when the preceding step fails or is unsuitable. The sequence was characterized retrospectively from the operative record and was not a prospectively defined protocol; post-extraction endoscopy was not performed by protocol. The algorithm is hypothesis-generating and has not been prospectively validated. Milking denotes delivery of the object to the anus by transmural manipulation without a deliberate incision in the bowel wall. TAMIS, transanal minimally invasive surgery; HALS, hand-assisted laparoscopic surgery.

**Figure 3:**
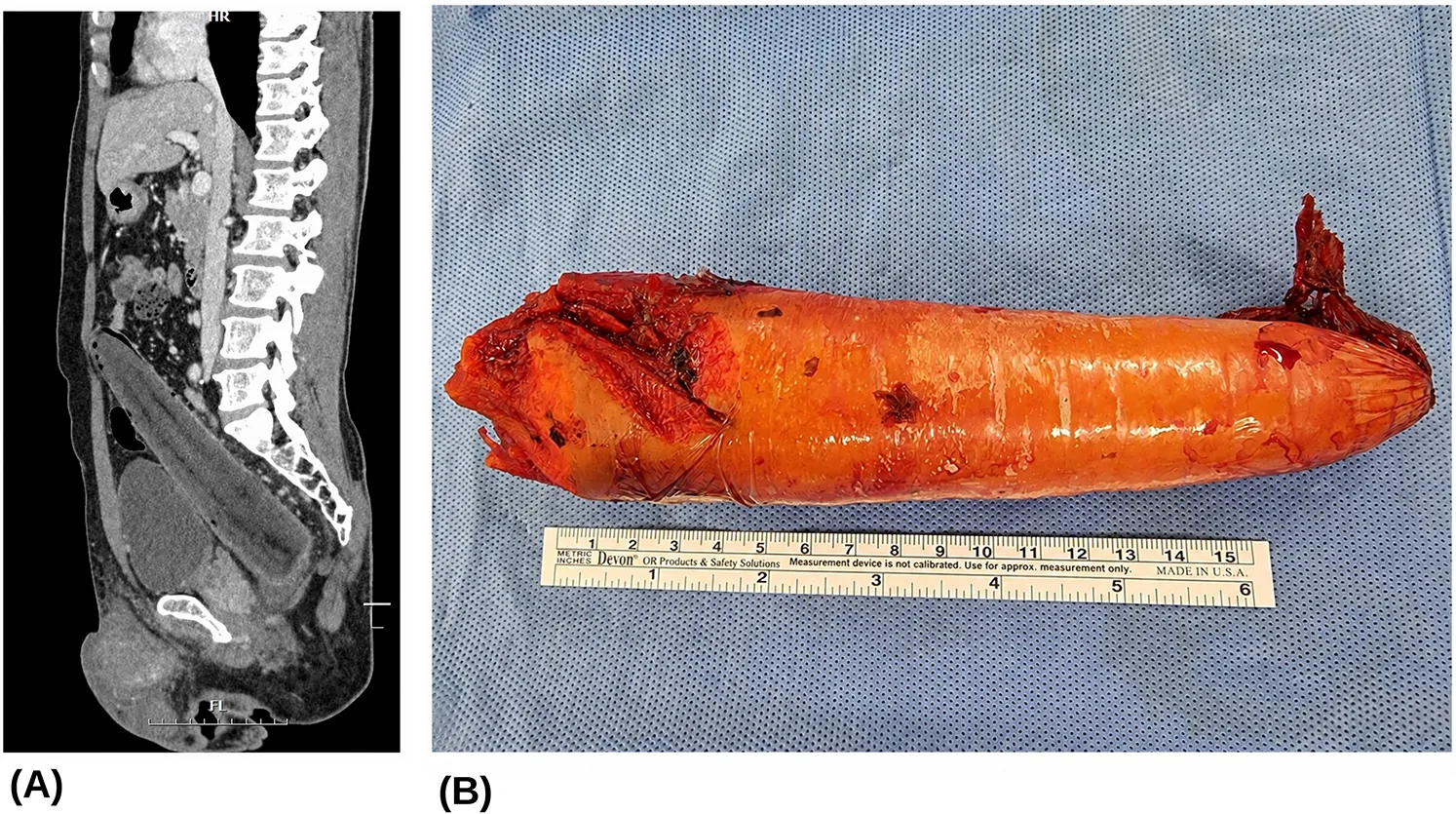
A retained rectal foreign body in a single patient. **(A)** Sagittal computed tomography shows a foreign body extending from the rectum to above the rectosigmoid junction. **(B)** The extracted specimen, a carrot 23 cm long and 5.5 cm in maximum diameter, was removed transanally; its soft, deformable consistency permitted transanal delivery. The ruler is uncalibrated and measurements are approximate.

### 4.2 Abdominal access is not synonymous with opening the bowel

Among the 42 series that explicitly distinguished milking from bowel opening, 64 of 181 abdominal procedures (35.4%) were completed by milking the object toward the anus without an enterotomy. In five series, every abdominal procedure was completed in this way (8, 10, 23–25). Two Russian series report the most striking version of this pattern: perforation occurred in patients who had been milked, and in both series, the perforation was repaired without the bowel ever being opened for extraction (24, 48). The equation of laparotomy with colotomy, and of colotomy with a stoma, is therefore not supported by the primary data.

Practice on diversion after colotomy is explicitly and symmetrically divided. Some groups state that colotomy is an absolute indication for a stoma (3, 23, 26); others state the opposite in equally direct terms (17, 27–29). Two series occupy the extremes of what was actually done: in one, both colotomies were closed primarily and no patient in the cohort received a stoma (15); in another, every bowel opening ended in a stoma (66). Neither position is supported by comparative data, and the variation appears to reflect surgeon and institutional preference.

The gap between recommendation and execution is documented within individual reports. Several series recommend milking in their discussion while reporting no instance of its use (15, 33, 67). One records the absence explicitly, stating that no attempt to milk the object distally was documented in any of the four cases in which the rectal wall was opened, while the same paper's discussion states that advancing the object distally is preferred over opening the bowel (21). Our two milking cases and two TAMIS cases are small in number, but they are case-level evidence that the intermediate steps are executable outside the single-center reports in which they were first described.

### 4.3 The range of abdominal intervention reflects cohort composition

Abdominal procedures were performed in 428 of 2,867 patients across the 59 series in which both figures could be determined, but the range was 0% to 75.0% and the distribution tracks cohort type rather than publication year. Four series reported no abdominal procedure at all (44, 45, 51, 64), while the highest proportions came from an operating-room-only registry analysis (65) and from two series in which patients presented predominantly with established complications (16, 41). All three of the highest values are from series published since 2018; an era effect, if present, is not visible against this variation.

The following three cohort types recur and are not interchangeable: whole-presentation cohorts that include bedside extraction, in which abdominal procedures are a small minority; operating-room or surgically referred cohorts, in which they are a substantial fraction; and administrative datasets, whose denominators are defined by coding. Our own 8 of 22 (36.4%) belongs to the second type and is comparable only with cohorts of that type. The structural consequence, i.e., that operative cohorts systematically omit the patients managed in the emergency department, was stated by Crass et al. in 1981 and restated in 2023 as a limitation shared by the two largest North American operative datasets (2, 69).

### 4.4 Post-extraction endoscopic assessment

Reported practice covers the entire possible range. Endoscopy after extraction was performed in every patient in several series (7, 8, 10, 14, 23, 69, 70) and in none in another (42), with intermediate rates of 79% (33), 76% (64), 45.5% (present series), 22.7% (15), and 7% (71). Where yield was also reported, injury was found in 20%–100% of those examined; our 60% (6 of 10) sits within this range.

More informative than the spread is that recommendations and practice diverge within single reports. One series concluded that post-extraction endoscopy should form part of a standardized pathway while reporting its own performance rate to be 22.7% (15). Another described flexible sigmoidoscopy as standard practice after removal, reported that it was performed in none of its 32 patients, and gave the following reason: endoscopy capacity in that trust was fully committed (42). A third reached the opposite conclusion from a 7% rate, arguing that routine examination is unnecessary (71). Consensus guidance reflects the same division, offering only a weak recommendation and describing the practice as debated (17).

Two observations follow. First, the injuries detected are overwhelmingly mucosal; submucosal integrity was preserved in all six of our patients with lacerations, and series reporting graded injury describe predominantly American Association for the Surgery of Trauma (AAST) grade I lesions (33, 88). Second, none of the series we reviewed states that a lesion found at post-extraction endoscopy altered management, although the majority do not address the question either way, nor did any such lesion alter management in our own cohort. The case for routine examination therefore rests on the detection of a rare transmural injury, and the evidence to support or refute a selective policy does not yet exist.

### 4.5 Sphincter injury and continence

Sphincter injury is recorded in this literature; its functional consequence almost never is. Two internal sphincter disruptions were reported without any statement of management or outcome (20). Moreover, an external sphincter laceration was repaired primarily with no follow-up after discharge (45), an internal sphincter tear was recorded without functional data (73), and one series reported decreased sphincter tone among its complications without giving a number (9).

The reason is documented and is not inattention. One group advised transrectal ultrasound and intervention for incontinence or stenosis after discharge and reported that every male patient declined (12). Another reported that 24 of 32 patients never attended outpatient review and stated that no standardized post-extraction assessment could therefore be recorded (30). Others described the same pattern, in one instance attributing it explicitly to the stigma attached to the presentation (26, 31, 32).

Where continence was captured, outcomes were generally favorable, although the numbers are small and the patients followed up may not be representative. The longest follow-up is a telephone interview at a median of 63 months with complete response, in which every patient was continent to gas, liquid, and solid stool (10). Others report no incontinence after anal dilatation (46, 69), no continence disturbance after stoma reversal (53), normal continence at 1 year (74), and preserved continence at discharge after primary repair of sphincter disruption (20). Our own observation, i.e., transient incontinence to liquid stool in 3 of 22 patients, all in the manual transanal group and all resolved within 2 months, is consistent with a pattern in which functional disturbance appears early and resolves, and long-term incontinence was not reported among the few patients followed.

Sphincterotomy remains contested. It is described as an aid to the extraction of large objects in several reports (7, 16, 23, 26, 46, 75) and as unavoidable for very large objects in one (49), while others advise against it on the grounds that the sphincter mechanism is at least partly destroyed (6, 27). Only one series we identified reports both how often it was performed and what followed: among 11 patients in whom transanal extraction was combined with posterior sphincterotomy, 5 reported anal pain on defecation, 4 reported incontinence to flatus, and 2 developed suppuration of the sphincterotomy wound; follow-up extended only to discharge at 4–7 days (43). That single dataset remains the only empirical counterweight we identified to a debate otherwise conducted on assertion.

### 4.6 What predicts the need for an abdominal approach?

Associations between object shape, consistency, or friability and the operative route were not formally tested in this cohort, and the material categories were assigned *post hoc* without reliability testing; the cases below are illustrative. Object dimensions did not distinguish our two groups. A high-lying carrot was delivered transanally by firm traction because it was soft and deformable, whereas a low-lying shampoo cap required a hand-assisted enterotomy because its wedge shape made transanal delivery impossible, and a friable porcelain cup fragmented during manipulation and was retrieved piecemeal.

The primary literature supports shape, consistency, and location over dimension. The most frequently cited analysis found that location predicted operative intervention whereas object size (*p* = 0.35) and dwell time (*p* = 0.95) did not (11). One series reported no significant difference between maximum object length and the method of successful retrieval and stated that shape mattered more than length (10). Another observed that larger objects were often easier to extract manually because they could be grasped, and reserved its concern for objects that were small, smooth, or fragile (89). A third listed nine factors that make retrieval difficult, including smooth surface, friability or rigidity, mucus and blood, sphincter spasm, mucosal edema, the sacral curve, the orientation of the blunt end, entrapment at the rectosigmoid junction or iliac spines, and negative pressure above the object, none of which is size (9). A 194-patient series describes consistency as critical and the presence of a point or edge as decisive in choosing between transanal and abdominal extraction, and notes the “plunger effect” produced by rigid, compact objects (3). Two further reports make the corresponding surgical point: advancement is inappropriate for sharp or broken objects, which should be extracted through an incision instead (21), and a broken glass object with fragments embedded in the mucosa was the single case in one series that required colotomy (19).

### 4.7 Internal consistency of the source literature

#### 4.7.1 Secondary sources

We did not use values reported in reviews or in the discussion sections of other papers, and this was an empirical rather than a precautionary decision. The most frequently cited analysis of predictors in this field has been misquoted in four subsequent publications, always in the same direction: findings reported as non-significant, e.g., object size and dwell time, reappear as predictors of failed transanal extraction (12, 28, 40, 90). The distortion compounds across citations: one report converts the 10 cm figure from object size into distance from the anal verge (12), and another adds a category, “hard or sharp objects,” that does not appear in the original analysis (90). Three publications quote the same source accurately, and each of them carries the negative finding on size forward as well (13, 17, 32). Summary tables show comparable problems. One review's tabulation of earlier series sums mutually exclusive categories, converting 98 abdominal procedures into 168 in one row, and misstates the abdominal and mortality counts of two further series (33). Because such tables are frequently the proximate source for subsequent reports, an error entered once propagates.

#### 4.7.2 Primary reports

Internal inconsistencies were also common in the included studies themselves, and we annotate the value adopted and the reason for each affected row in Supplementary Table S6. They fall into the following recurring types: percentages that do not correspond to their numerators (3, 43, 66); counts that differ between text and tables (12, 14, 45); abstracts that differ from results (15, 67, 77); and denominators that shift between patients, presentations, and objects within a single paper (9, 15, 22, 91, 92). In one series, the reported management of an individual case is incompatible with its reported outcome (77). Only two of these affected our primary endpoints once the adopted value was identified; the remainder concern secondary items. The point is not that this literature is unusually careless—series of this size, written from emergency records, are prone to exactly these errors—but that the discrepancies are invisible in a summary table and recoverable only from the full text.

#### 4.7.3 Bibliographic drift

Citation details degrade in the same way. The page range of one series is given as 606–611 in the original and as 607–612 in two subsequent citations (16, 91); the page range of another differs between the original article and its database record; the first author of a 1977 report is misspelled identically in two unrelated papers; and the reference list of one recent series contains a title from an unrelated field. These are minor individually, but together they made author- and page-level verification a necessary step rather than a formality, and they are the reason we report the bibliographic source used for reports obtained through archival or other supplementary search routes.

### 4.8 A note on item-level appraisal

Reporting the JBI checklist item by item rather than as a composite score makes the underlying reporting patterns visible and avoids treating unequal checklist items as interchangeable. The final 60-study appraisal shows that uncertainty arose mainly from reporting of sampling rather than from identification of the condition: consecutive inclusion was unclear in 36 studies and complete inclusion in 41, whereas valid identification methods were judged adequate in 53 and demographic reporting in all 60. Item 1 was judged solely on the clarity of each original series’ inclusion criteria and was not used as a proxy for whether a mixed cohort was separable under this review's eligibility criteria. The two reports added during the final archival closure sweep were appraised with the same item-level rules as the earlier reports.

### 4.9 Limitations

#### 4.9.1 The cohort

Patients whose foreign body was removed at the bedside or in the endoscopy suite were not captured by the operative registries used for case identification, so the series represents surgically managed foreign bodies and the 36.4% abdominal proportion is a denominator within a surgical cohort rather than a population operative rate. The series is retrospective and comprises 22 patients, which precludes multivariable analysis; between-group comparisons are exploratory and unadjusted, and because object location and perforation both informed the choice of approach, the associations reported are not independent predictors of it. Object material was categorized *post hoc* without reliability testing and was not used for formal testing.

#### 4.9.2 Functional and endoscopic endpoints

Continence was assessed by history and digital examination rather than by manometry, endoanal imaging, or a validated score, so transient incontinence resolving within 2 months cannot exclude subclinical sphincter injury. Minimum follow-up was 2 months—longer than most series in this field, but far short of the interval at which the only long-term continence data were obtained—and cannot address late stricture, fistula, or delayed deterioration. Post-extraction endoscopy was performed at the surgeon's discretion in 10 of 22 patients, so the 60% yield derives from a selected subgroup and probably overestimates the yield of routine examination.

#### 4.9.3 The index techniques

TAMIS was used in two patients and laparoscopic or hand-assisted milking in another two. These cases demonstrate that the intermediate steps are executable within a stepwise strategy; they cannot establish indications, comparative safety, or thresholds for escalation, and the algorithm remains hypothesis-generating and has not been prospectively validated.

#### 4.9.4 Review protocol

The protocol was registered on the Open Science Framework during this revision, after the original submission and after extraction had begun; retrospective registration provides transparency but not protection against *post hoc* changes. The minimum series size was raised from three to five patients after screening had begun, because three- and four-patient series could not reliably be distinguished from case reports; the studies excluded by this change are listed individually in the supplementary exclusion table, and a sensitivity analysis retaining the five that contained a derivable eligible subgroup is provided, in which the abdominal procedure proportion changed by 0.05 percentage points and no direction of finding was reversed. Screening of the rebuilt search was performed independently and in duplicate by two reviewers, as in the original submission; the search itself was re-run in full.

#### 4.9.5 Synthesis

Included series span 1976–2026 and differ in cohort definition, imaging availability, coding practice, and the meaning of “operative”; no meta-analysis was performed, and the aggregate figures are crude sums across studies with different denominators rather than pooled estimates. The proportion of abdominal procedures completed by milking is derived only from the 42 series that recorded the distinction; series that record it may be those in which milking was performed, and the direction of the resulting bias cannot be determined. Nine included studies combined inserted retained objects with ingestion, iatrogenic migration, or trauma without a separable subgroup, so the aggregate denominator contains an unknown number of patients who would not meet our insertion criterion; a review-level sensitivity analysis excluding these nine changes the aggregate proportion of abdominal procedures from 428/2,867 (14.9%) to 303/1,908 (15.9%) (Supplementary Table S4). Khan (2021) remains in this sensitivity analysis because its eligible 15-patient subgroup was separable from the parent cohort. Outcome reporting was incomplete and is unlikely to be missing at random: mortality was reported in 54 of 60 series, stoma formation in 52, and colotomy counts in 42, and no values were imputed. Abandoning a composite quality score removes any basis for weighting studies, so all included series contribute equally to the descriptive figures.

Two source-level qualifications are disclosed explicitly: the eligible subgroup size for Ayantunde and Oke (2006) was confirmed from a subsequent report by the same authors because the original subgroup table was unavailable, and the milking classification for three of four abdominal procedures in Trenin (2005) was supported by clarification from the corresponding author of a later Russian series. Trenin (2005) is additionally removed in the sensitivity analysis excluding non-separable mixed cohorts.

#### 4.9.6 Coverage and overlap

The review included reports identified outside the four database search outputs through citation searching and targeted journal searches. Five of the nine reports not retrieved were in a Turkish national colorectal journal whose earlier volumes are neither indexed nor digitized; one, +Korkut et al. (1995), is described in its title as a retrospective five-patient series and would probably have been eligible. Chinese-language databases were not searched; a 2022 systematic review retrieved 86 records from four Chinese databases against 73 from English-language sources on this topic. Four pairs of reports were treated as linked publications on the basis of institution, study period, and authorship rather than patient identifiers, and three series from one city with overlapping study periods but different institutions were retained separately, so patient overlap between them cannot be formally excluded. Non-English full texts were extracted with translation assistance and numeric tables cross-checked against the original-language text, which will have increased the proportion of “unclear” appraisal judgments for those reports.

#### 4.9.7 Reporting bias

This condition is under-reported at presentation, and published series come disproportionately from institutions with an interest in it. Emergency-department surveillance data suggest a female proportion of approximately one in five, whereas operative series report 76%–99% male; our cohort cannot distinguish between differential progression to surgery and differential reporting.

## 5 Conclusion

In this multicenter operating-room series and an accompanying synthesis of 60 published series, a stepwise, organ-preserving approach was feasible in the majority of patients requiring operative extraction of a rectal foreign body: escalating from transanal and TAMIS extraction, to laparoscopic or hand-assisted milking, and to enterotomy, with resection and diversion reserved for perforation with severe bowel-wall injury, contamination, or non-viable tissue. The published data show that abdominal access does not entail opening the bowel in approximately one-third of abdominal procedures where the distinction is recorded, and that colotomy was closed primarily without diversion in selected reported cases. Object location was associated with the operative route in our cohort, whereas object size was not; case observations and prior reports suggest that shape and consistency also matter, but this was not formally tested here. Functional outcomes after extraction remain almost unmeasured for a documented reason, namely, patients characteristically decline follow-up, and the few series that have obtained them report preserved continence. A prospective study with a defined follow-up protocol and validated continence assessment would address the single largest gap in this literature.

## Data Availability

The raw data supporting the conclusions of this article will be made available by the authors, without undue reservation.
