# Supplementary material for: Stepwise surgical management of rectal foreign bodies: a multicenter operative case series and systematic review
Source: Front Surg. 2026 Oct 8;13:1933279. doi: 10.3389/fsurg.2026.1933279 (PMC13687044; doi:10.3389/fsurg.2026.1933279)
Supplement: Supplementary file 1 [file Datasheet1.pdf]

Supplementary Material

Supplementary Table S1. Patient-level summary of all 22 cases

"Initial attempt" is the first approach undertaken in the operating room; "Completed approach" is the approach by which extraction was completed. Two minimally invasive attempts were converted (case 16, laparoscopic to hand-assisted; case 19, laparoscopic to open). Location is given relative to the rectosigmoid junction. Milking denotes delivery of the object to the anus by transmural manipulation without a deliberate incision in the bowel wall and is not counted as opening the bowel for extraction; case 19 underwent lavage and primary repair of a pre-existing contained perforation and is likewise not counted among the deliberate bowel openings. Material categories were assigned post hoc by the investigators from the object recorded at operation. HALS, hand-assisted laparoscopic surgery; LAR, low anterior resection; LOS, length of stay; NA, not measured; TAMIS, transanal minimally invasive surgery.

#	Age/Sex	Object	Material category	Diameter × length (cm)	Location	Free air	Initial attempt	Completed approach	Procedure	Bowel wall	Stoma	Clavien–Dindo	LOS (d)
1	34/M	Adult toy	Rigid/non-brittle	4 × 18	Below	No	Transanal	TAMIS	TAMIS extraction	Not opened	No	0	1
2	58/M	Porcelain cup	Rigid/brittle	5 × 16	Below	No	Transanal (failed)	Open	Enterotomy, piecemeal	Extraction enterotomy, primary closure	No	0	8
3	41/M	Cucumber	Organic/deformable	3.5 × 22	Below	No	Transanal	Transanal	Manual/instrumental	Not opened	No	0	1
4	56/M	Shampoo cap (dome)	Rigid plastic/wood	NA	Above	No	Transanal (failed)	HALS	HALS enterotomy + primary repair	Extraction enterotomy, primary closure	No	II	9
5	29/M	Glass cup	Rigid/brittle	5.5 × 9	Below	No	Transanal	Transanal	Manual/instrumental	Not opened	No	0	2
6	52/F	Adult toy	Rigid/non-brittle	2 × 12	Below	No	Transanal	Transanal	Manual/instrumental	Not opened	No	I	2
7	45/M	Metal handle	Rigid/non-brittle	3 × 17	Below	No	Transanal	Transanal	Manual + Foley traction	Not opened	No	0	1
8	36/M	Electric toothbrush	Rigid/non-brittle	3.2 × 19	Below	No	Transanal	Transanal	Manual/instrumental	Not opened	No	0	2
9	61/M	Large carrot	Organic/deformable	5.5 × 23	Above	No	Transanal (GA)	Transanal	Manual (soft, milked distally)	Not opened	No	I	6
10	39/M	Plastic bottle cap	Rigid plastic/wood	4.5 × 5	Below	No	Transanal	Transanal	Manual + endoscopic snare	Not opened	No	0	1
11	74/F	Adult toy	Rigid/non-brittle	2.5 × 30	Above	No	Transanal	Transanal	Manual/instrumental	Not opened	No	I	3
12	48/M	Glass bottle	Rigid/brittle	6.5 × 18	Above	Yes	Open	Open	Resection + Hartmann	Resection	Yes	IIIb	14
13	33/M	Toy (banana)	Rigid/non-brittle	4 × 21	Below	No	Transanal	Transanal	Manual/instrumental	Not opened	No	0	1
14	56/F	Anal plug	Elastomeric	3.5 × 5	Above	No	Laparoscopic	Laparoscopic	Laparoscopic milking	Not opened (milking)	No	0	2
15	43/M	Aluminum can	Rigid/non-brittle	5.8 × 11	Below	No	Transanal	Transanal	Manual/instrumental	Not opened	No	I	2

#	Age/Sex	Object	Material category	Diameter × length (cm)	Location	Free air	Initial attempt	Completed approach	Procedure	Bowel wall	Stoma	Clavien–Dindo	LOS (d)
16	69/M	Wooden umbrella handle	Rigid plastic/wood	3 × 13	Above	No	Laparoscopic → HALS	HALS	HALS milking	Not opened (milking)	No	I	12
17	25/M	Soap case	Rigid plastic/wood	4.2 × 8	Below	No	Transanal	TAMIS	TAMIS extraction	Not opened	No	0	1
18	62/M	Massage device	Rigid/non-brittle	3.8 × 23	Below	No	Transanal	Transanal	Manual/instrumental	Not opened	No	0	2
19	71/M	Wooden stick	Rigid plastic/wood	3 × 28	Above	Yes	Laparoscopic → open	Open	Lavage + primary repair	Repair of pre-existing perforation	No	II	9
20	50/M	Rubber ball	Elastomeric	10 (sphere)	Above (to descending colon)	Yes	Open	Open	Bowel rupture, Hartmann	Resection	Yes	II	9
21	38/M	Silicone plug	Elastomeric	4.5 × 12	Below	No	Transanal	Transanal	Manual/instrumental	Not opened	No	0	1
22	64/M	Shampoo container	Rigid plastic/wood	6.8 × 17	Above	Yes	Open	Open	LAR + diverting ileostomy	Resection	Yes	IIIb	16

Derived totals — transanal or TAMIS 14 (transanal 12, TAMIS 2); abdominal 8 (open 5, HALS 2, laparoscopic 1); milking without deliberate bowel incision 2; deliberate bowel opening for extraction 5 (extraction enterotomy with primary closure 2, resection 3); repair of a pre-existing contained perforation 1; stoma 3; perforation 4; in-hospital death 0.

All 22 patients had a minimum clinical follow-up of two months. Post-extraction endoscopy and continence outcomes are reported in aggregate in the main text because patient-level allocation was not prespecified for this table.

Supplementary Table S2. Reports excluded at full-text assessment, with reasons (n = 67)

Pathway denotes whether the report was identified in the four original database exports (Database) or only through backward citation searching and targeted journal or archive searching (Other methods). Database pathway n = 44; other-methods pathway n = 23.

#	Report	Reason for exclusion	Pathway	Note
1	Kurer MA, Davey C, Khan S et al. Colorectal foreign bodies: a systematic review. Colorectal Dis. 2010;12(9):851-61. doi: 10.1111/j.1463-1318.2009.02109.x	Review, guideline, or textbook without original patient data	DB	
2	Ploner M, Gardetto A, Ploner F et al. Foreign rectal body - Systematic review and meta-analysis. Acta Gastroenterol Belg. 2020;83(1):61-65.	Review, guideline, or textbook without original patient data	DB	
3	Yang Z, Xin P, Zhou S et al. Systematic review of rectal foreign bodies in older men: humanistic care and a novel challenge for society. Ann Transl Med. 2022;10(4):164. doi: 10.21037/atm-22-103	Review, guideline, or textbook without original patient data	DB	
4	Pandey S, Chhetri SK, Agrawal A, et al. Rectal foreign body in South Asia: a systematic review. Yemen J Med. 2026;5(1):86-94. doi:10.63475/yjm.v5i1.0344	Review, guideline, or textbook without original patient data	Other	
5	Goldberg JE, Steele SR. Rectal foreign bodies. Surg Clin North Am. 2010;90(1):173-84, Table of Contents. doi: 10.1016/j.suc.2009.10.004	Review, guideline, or textbook without original patient data	DB	
6	Cologne KG, Ault GT. Rectal foreign bodies: what is the current standard?. Clin Colon Rectal Surg. 2012;25(4):214-8. doi: 10.1055/s-0032-1329392	Review, guideline, or textbook without original patient data	DB	
7	Tarasconi A, Perrone G, Davies J et al. Anorectal emergencies: WSES-AAST guidelines. World J Emerg Surg. 2021;16(1):48. doi: 10.1186/s13017-021-00384-x	Review, guideline, or textbook without original patient data	DB	
8	Cleary RK, Pomerantz RA, Lampman RM. Colon and rectal injuries. Dis Colon Rectum. 2006;49(8):1203-22.	Review, guideline, or textbook without original patient data	Other	
9	Ebrahim M, Perdawood SK. Fremmedlegeme i rectum. Ugeskr Laeger. 2018;180(36):V11170866. Danish.	Review, guideline, or textbook without original patient data	DB	
10	Давидов МИ, Владимирский ЕВ, Никонова ОЕ. [Classification, clinical features and diagnosis of foreign bodies of the rectum]. Дневник Казанской медицинской школы. 2018;I(XIX):6-11. Russian.	Review, guideline, or textbook without original patient data	Other	
11	Wigle RL. Emergency department management of retained rectal foreign bodies. Am J Emerg Med.	Review, guideline, or textbook without original patient data	DB	

#	Report	Reason for exclusion	Pathway	Note
	1988;6(4):385-9. doi: 10.1016/0735-6757(88)90163-5			
12	Busch DB, Starling JR. Rectal foreign bodies: case reports and a comprehensive review of the world's literature. Surgery. 1986;100(3):512-9.	Review, guideline, or textbook without original patient data	DB	
13	Bokhan KL. [Rectal foreign bodies]. Khirurgiia (Mosk). 2000;(6):63-6.	Review, guideline, or textbook without original patient data	DB	
14	Rocklin MS, Apelgren KN. Colonoscopic extraction of foreign bodies from above the rectum. Am Surg. 1989;55(2):119-23.	Review, guideline, or textbook without original patient data	DB	
15	Аминев АМ. [Manual of proctology]. Куйбышев; 1965. Russian. (monograph)	Review, guideline, or textbook without original patient data	Other	Russian-language monograph
16	Pavlidis T, Marakis G, Psarras K, et al. The role of endoscopy for foreign bodies in the rectum. Internet J Surg. 2005;8(1).	Case report, or series of fewer than five patients	Other	
17	Atila K, Sökmen S, Astarcıoğlu H, Canda E. [Foreign body in the rectum: report of four cases]. Ulus Travma Derg. 2004;10(4):253-6. Turkish.	Case report, or series of fewer than five patients	DB	
18	Sangar P, Henry G, Sood S. Foreign bodies in the rectum: report of a case series and review of the literature. Sri Lanka J Surg. 2013;31(2):27-30.	Case report, or series of fewer than five patients	Other	
19	Karateke F, Das K, Ozyazici S et al. Anorectal Injuries due to Foreign Bodies: Case Reports and Review of the Management Options. Case Rep Surg. 2013;2013:809592. doi: 10.1155/2013/809592	Case report, or series of fewer than five patients	DB	
20	Tofigh AM, Salimi S, Nematihonar B et al. Foreign bodies in the rectum: Three case reports of sexual violence. Ann Med Surg (Lond). 2022;78:103695. doi: 10.1016/j.amsu.2022.103695. eCollection 2022 Jun doi:10.1016/j.amsu.2022.103695	Case report, or series of fewer than five patients	DB	
21	Memon JM, Memon NA, Khatri MK, et al. Rectal foreign body: not a rarity. Gomal J Med Sci. 2007;5(2):72-4.	Case report, or series of fewer than five patients	Other	
22	Akbulut S, Yılmaz F. [Foreign body in the rectum: a case report and review of the literature]. Kolon Rektum Hast Derg. 2011;21(4):182-6. Turkish.	Case report, or series of fewer than five patients	Other	
23	Bilge Z, Öztaş E, Özderin Özin Y, Öztaş E, Dişibeyaz S. [Foreign body in the rectum: an intrauterine device]. Endoskopi Gastrointestinal. 2015;23(2):62-3. doi:10.17940/endoskopi.468352. Turkish.	Case report, or series of fewer than five patients	Other	Case report of an intrauterine device that had migrated into the rectum; not a per-anum insertion
24	Oruç C, Uğur M, Akküçük S, Çelikel A, Kılıç E, Ortanca İ. [Inorganic foreign body in	Case report, or series of fewer than five patients	Other	

#	Report	Reason for exclusion	Pathway	Note
	rectum: case report]. Int J Basic Clin Med. 2015;3(3):131-4. Turkish.			
25	Coşkun İ, Taş R, Uzunköy A. [Foreign body in the rectum: a stone (a case report)]. Kolon ve Rektum Hastalıkları Dergisi. 1992;2:194-6. Turkish.	Case report, or series of fewer than five patients	Other	Full text not obtained; the title states that the report is a single-case report
26	Yılmaz Z, Pekrü İ. [Foreign body in the rectum: a tea glass]. Kolon ve Rektum Hastalıkları Dergisi. 1993;3:102-3. Turkish.	Case report, or series of fewer than five patients	Other	Full text not obtained; Atila 2004 and Batmaz 2002 cite this report as a single case of one retained tea glass, and the title itself names a single object
27	Стяжкина СН, и др. [Foreign bodies of the rectum]. Colloquium-Journal. 2020;13-2(65):51-3. Russian.	Case report, or series of fewer than five patients	Other	
28	Бутырский АГ, Шерендак СА, Аметов АУ, Новодранов ВВ. [A rare case of a foreign body of the colon]. Таврический медико-биологический вестник. 2020;23(1):124-7. doi:10.29039/2070-8092-2020-23-1-124-127. Russian.	Case report, or series of fewer than five patients	Other	previously recorded in error as 2022
29	Gajjar RA, Gupta PB. Foreign body in the rectum: A challenge for the emergency physician. J Family Med Prim Care. 2016;5(2):495-497. doi: 10.4103/2249-4863.192381	Case report, or series of fewer than five patients	DB	
30	Ryoo SB, Oh HK, Ha HK et al. Operative treatment with a laparotomy for anorectal problems arising from a self-inserted foreign body. J Korean Soc Coloproctol. 2012;28(1):56-60. doi: 10.3393/jksc.2012.28.1.56	Case report, or series of fewer than five patients	DB	
31	Nepal A, Maharjan S, Chalise A et al. Rectal Foreign Body: A Case Report. JNMA J Nepal Med Assoc. 2022;60(256):1049-1051. doi: 10.31729/jnma.7905	Case report, or series of fewer than five patients	DB	
32	Hassine HB, Chaka A, Ouertani F et al. Management of a rectal entrapped foreign body: a case report. Pan Afr Med J. 2024;48:162. doi: 10.11604/pamj.2024.48.162.43548. eCollection 2024 doi:10.11604/pamj.2024.48.162.43548	Case report, or series of fewer than five patients	DB	
33	Chen L, Zhang Y, Zhou W et al. Case Report: Transanal extraction combined with wound management for a broken glass foreign body and associated rectal injuries. Front Surg. 2026;13:1803580. doi: 10.3389/fsurg.2026.1803580. eCollection 2026 doi:10.3389/fsurg.2026.1803580	Case report, or series of fewer than five patients	DB	
34	Gupta SK, Katiyar VK, Sharma S et al. Minimally invasive techniques as adjuncts in low- versus high-lying retained rectal foreign bodies of autoerotic nature in young men: a tailored management algorithm with	Case report, or series of fewer than five patients	DB	

#	Report	Reason for exclusion	Pathway	Note
	two contrasting case reports from India. J Trauma Inj. 2024;37(3):238-242. doi: 10.20408/jti.2024.0003.20408/jti.2024.0003			
35	Han HJ, Joung SY, Park SH et al. Transanal rectal foreign body removal using a SILS port. Surg Laparosc Endosc Percutan Tech. 2012;22(3):e157-8. doi: 10.1097/SLE.0b013e31824a54aa	Case report, or series of fewer than five patients	DB	
36	Bak Y, Merriam M, Neff M et al. Novel approach to rectal foreign body extraction. JSLS. 2013;17(2):342-5. doi: 10.4293/108680813X13654754534233	Case report, or series of fewer than five patients	DB	
37	Elias B, Debs T, Hage S et al. Single incision laparoscopic surgery technique for transanal removal of rectal foreign body. J Surg Case Rep. 2014;2014(3):rju022. doi: 10.1093/jscr/rju022	Case report, or series of fewer than five patients	DB	
38	Cawich SO, Mohammed F, Spence R et al. Colonic Foreign Body Retrieval Using a Modified TAMIS Technique with Standard Instruments and Trocars. Case Rep Emerg Med. 2015;2015:815616. doi: 10.1155/2015/815616	Case report, or series of fewer than five patients	DB	
39	Berghoff KR, Franklin ME Jr. Laparoscopic-assisted rectal foreign body removal: report of a case. Dis Colon Rectum. 2005;48(10):1975-7. doi: 10.1007/s10350-005-0117-6	Case report, or series of fewer than five patients	DB	
40	Rispoli G, Esposito C, Monachese TD et al. Removal of a foreign body from the distal colon using a combined laparoscopic and endoanal approach: report of a case. Dis Colon Rectum. 2000;43(11):1632-4. doi: 10.1007/BF02236754	Case report, or series of fewer than five patients	DB	
41	Bulte JP, Clermonts SHEM, Zimmerman DDE. Antegrade impalement of the rectum by a foreign body: a case report of a successful transanal extraction using the transanal single port (TAMIS) technique. Tech Coloproctol. 2019;23(2):175-178. doi: 10.1007/s10151-019-1929-z	Case report, or series of fewer than five patients	DB	
42	Chilakamarry S, Klipfel AA. Novel Approach to Removal of Rectal Foreign Bodies. Dis Colon Rectum. 2021;64(6):e368-e370. doi: 10.1097/DCR.0000000000002048	Case report, or series of fewer than five patients	DB	
43	Kasotakis G, Roediger L, Mittal S. Rectal foreign bodies: A case report and review of the literature. Int J Surg Case Rep. 2012;3(3):111-5. doi: 10.1016/j.ijscr.2011.11.007	Case report, or series of fewer than five patients	DB	
44	Waraich NG, Hudson JS, Iftikhar SY. Vibrator-induced fatal rectal	Case report, or series of fewer than five patients	DB	

#	Report	Reason for exclusion	Pathway	Note
	perforation. N Z Med J. 2007;120(1260):U2685.			
45	Weissberg D. Foreign bodies in the gastro-intestinal tract. S Afr J Surg. 1991;29(4):150-3.	Mixed cohort; eligible inserted subgroup not separable and prespecified retention criteria not met	DB	previously recorded in error as 1981
46	Bloom RR, Nakano PH, Gray SW et al. Foreign bodies of the gastrointestinal tract. Am Surg. 1986;52(11):618-21.	Mixed cohort; eligible inserted subgroup not separable and prespecified retention criteria not met	DB	
47	Bathó G, Szántó L. [Foreign bodies in the rectum at our department during the last ten years]. Magy Seb. 2000;53(4):180-2.	Mixed cohort; eligible inserted subgroup not separable and prespecified retention criteria not met	DB	
48	Koplatadze AM, Bondarev IuA. [Injuries and foreign bodies of the rectum]. Khirurgiia (Mosk). 1990;(9):53-6.	Mixed cohort; eligible inserted subgroup not separable and prespecified retention criteria not met	DB	Bibliographically distinct from Koplatadze 1995
49	Koplatadze AM, Kim SD, Kamaeva DK. [Diagnosis of rectal trauma and foreign bodies and treatment tactics]. Ros Zh Gastroenterol Gepatol Koloproktol. 1995;5(1):73-7. Russian.	Mixed cohort; eligible inserted subgroup not separable and prespecified retention criteria not met	Other	Bibliographically distinct from Koplatadze 1990. Potential cohort overlap with the included Kim SD 2008 series could not be determined from the available reports
50	Marti MC, Morel P, Rohner A. Traumatic lesions of the rectum. Int J Colorectal Dis. 1986;1(3):152-4. doi:10.1007/BF01648441.	Mixed cohort; eligible inserted subgroup not separable and prespecified retention criteria not met	Other	Series of traumatic rectal injuries (n=22) of mixed mechanism: gunshot 2, sexual trauma 8, road traffic 5, impalement 5, polypectomy 2
51	Loria A, Marianetti I, Cook CA et al. Epidemiology and healthcare utilization for rectal foreign bodies in United States adults, 2012-2021. Am J Emerg Med. 2023;69:76-82. doi: 10.1016/j.ajem.2023.03.041	Extraction route or operative outcome not derivable	DB	
52	Sheets NW, Waldrop I, Carpenter WC et al. Rectal Foreign Bodies: A 10-Year Review of the National Electronic Injury Surveillance System. Cureus. 2023;15(7):e41471. doi: 10.7759/cureus.41471. eCollection 2023 Jul doi:10.7759/cureus.41471	Extraction route or operative outcome not derivable	DB	
53	Bhasin S, Williams JG. Rectal foreign body removal: increasing incidence and cost to the NHS. Ann R Coll Surg Engl. 2021;103(10):734-737. doi: 10.1308/rcsann.2020.7129	Extraction route or operative outcome not derivable	DB	
54	Schellenberg M, Brown CVR, Trust MD et al. Rectal Injury After Foreign Body Insertion: Secondary Analysis From the AAST Contemporary Management of Rectal Injuries Study Group. J Surg Res. 2020;247:541-546. doi: 10.1016/j.jss.2019.09.048	Extraction route or operative outcome not derivable	DB	
55	Сергацкий КИ, Логачев ДА, Никольский ВИ, Митрошин АН, Феоктистов ЯЕ, Кошелькова ДА. [Colotomy in the extraction of foreign bodies of the colon: how to finish the operation?]. Актуальные проблемы медицины. 2025;48(4):536-44. doi:10.52575/2687-0940-	Extraction route or operative outcome not derivable	Other	Cohort defined by receipt of colotomy; no denominator of all presentations

#	Report	Reason for exclusion	Pathway	Note
	2025-48-4-536-544. Russian.			
56	Byard RW, Eitzen DA, James R. Unusual fatal mechanisms in nonasphyxial autoerotic death. Am J Forensic Med Pathol. 2000;21(1):65-8. doi: 10.1097/00000433-200003000-00012	Extraction route or operative outcome not derivable	DB	title contains no foreign-body or rectal term, so the record was initially missed on screening of the export
57	Lo PC, Chen JS, Lin PW, et al. A multidisciplinary algorithm for retained colorectal foreign body. J Soc Colon Rectal Surgeon (Taiwan). 2022.	Duplicate or linked publication of an included cohort	Other	Cheung 2019 was retained as the primary report
58	Chowdhury A, Havelka E, O'Farrell E, Pangeni A, Shrestha AK. A retrospective study: rectal foreign bodies, enter at your own risk. Br J Surg. 2022;109(Suppl 5):znac247.107.	Duplicate or linked publication of an included cohort	DB	Conference abstract of the East Kent cohort; O'Farrell 2022 was retained as the primary full report
59	Rodríguez-Hermosa JI, Codina-Cazador A, Alayrach Vilella J, Sirvent JM, Roig J, Farrés R, et al. [Foreign bodies in the rectum and sigmoid colon]. Cir Esp. 2001;69(4):404-7. doi:10.1016/S0009-739X(01)71764-2. Spanish.	Duplicate or linked publication of an included cohort	DB	
60	Нурматова МА, Даминова НМ. [Surgical treatment of rectal foreign bodies]. Здравоохранение Таджикистана. 2015;(2):37-40. Russian.	Duplicate or linked publication of an included cohort	Other	linked publication of the Daminova 2014 cohort
61	Ozbilgin M, Arslan B, Yakut MC et al. Five years with a rectal foreign body: A case report. Int J Surg Case Rep. 2015;6C:210-3. doi: 10.1016/j.ijscr.2014.11.053	Duplicate or linked publication of an included cohort	DB	
62	Mulita F, Theofanis G, Tchabashvili L et al. Rectal foreign bodies: retained orange. Prz Gastroenterol. 2021;16(4):392-393. doi: 10.5114/pg.2021.105409	Duplicate or linked publication of an included cohort	DB	
63	Sohn N, Weinstein MA, Gonchar J. Social injuries of the rectum. Am J Surg. 1977;134(5):611-2.	No retained foreign body	Other	
64	Choi KD. [Foreign bodies in the anorectum: report of five cases and a review of the literature]. J Korean Soc Coloproctol. 1990;6(1):55-62. Korean.	No retained foreign body	Other	all five cases were ingested or impalement-related
65	Ikeda N, Hulewicz B, Knight B et al. Homicide by rectal insertion of a walking stick. Nihon Hoigaku Zasshi. 1991;45(4):341-4.	No retained foreign body	DB	
66	Albayrak D, İbiş AC, Hatipoğlu AR, İrfanoğlu ME. [Foreign bodies in the rectum: a report of three cases]. Trakya Univ Tıp Fak Derg. 2007;24(3):240-3. Turkish.	Case report, or series of fewer than five patients	Other	Three patients, all managed transanally
67	Akkaya N. [Anorectal emergencies]. In: [Gastrointestinal system emergencies]. Turkish.	Review, guideline, or textbook without original patient data	Other	Textbook chapter without original patient data

Exclusions by reason and pathway

Reason	Database	Other methods	Total
Review, guideline, or textbook without original patient data	11	5	16
Case report, or series of fewer than five patients	19	11	30
Mixed cohort; eligible inserted subgroup not separable and prespecified retention criteria not met	4	2	6
Extraction route or operative outcome not derivable	5	1	6
Duplicate or linked publication of an included cohort	4	2	6
No retained foreign body	1	2	3
Total	44	23	67

Supplementary Table S3. Corrections and methodological changes made during revision

The original submission (manuscript ID 1933279) was re-audited against the primary reports and the case-level source data during revision. To preserve provenance, this table separates **values that were actually present in the original submission** from **values introduced during the expanded revision and subsequently corrected**. Values that first appeared only during revision are therefore not labelled as original-submission values.

A. Corrections to values reported in the original submission

#	Study / field	Original submission	Final revised value	Basis for correction
1	Present study — case 9 object dimensions and Table 2 size comparison	Figure 3: carrot approximately 17 × 5 cm; Table 2 diameter p = 0.313; transanal/TAMIS length 17 (5–30) cm, p = 0.737	Case 9: 23 × 5.5 cm; Table 2 diameter p = 0.331; transanal/TAMIS length 17.5 (5–30) cm, p = 0.654	Case-level dimensions were re-verified and the Mann–Whitney comparisons were recalculated.
2	Present study — abdominal procedure decomposition	"Enterotomy was required in 6 patients"; two additional proximal objects were milked distally without enterotomy	Abdominal approach 8: milking without deliberate bowel incision 2; extraction enterotomy with primary closure 2; resection 3; repair of a pre-existing contained perforation without extraction enterotomy 1	Revision distinguished deliberate bowel opening for extraction from resection and from repair of a pre-existing perforation.
3	Barone 1976 — N / abdominal / stoma	28 / 5 / NR	23 / 0 / 0	Five patients in whom the object had already passed before presentation were removed from the retained-object denominator; operative values were re-verified from the primary report.
4	Eftaiha 1977 — N / abdominal / stoma	31 / NR / 0	30 / 3 / 1	Primary-report re-verification.
5	Barone 1983 — N / abdominal / stoma / death	36 / 10 / NR / NR	35 / 1 / 1 / 0	Primary-report re-verification using the retained-object cohort and operative details.
6	Cohen 1996 — abdominal procedures	6	0	Primary-report re-verification; the original extraction had conflated operative categories.
7	Lake 2004 — stoma	NR	2	Primary-report re-verification.
8	Rodríguez-Hermosa 2007 — abdominal / stoma	NR / NR	2 / ≤2	The two laparotomies attributable to the 16-patient anally introduced subgroup were separable; exact stoma attribution within that subgroup remained limited.
9	Safioleas 2009 — stoma	NR	≥1	Primary-report re-verification; at least one diversion was directly supportable, but the exact total could not be resolved confidently.
10	Volpi 2012 — N / abdominal / stoma / death	NR / NR / NR / NR	10 / 2 / 1 / 0	Values were present in the primary report but had not been extracted in the original submission.
11	Coskun 2013 — stoma	NR	1	Primary-report re-verification.
12	Khan 2021 — N / abdominal / stoma / death	NR / NR / NR / NR	15 / 10 / 0 / 0	Primary-report re-verification showed that the eligible 15-patient subgroup was separable from the 22-patient parent cohort.
13	Zhang 2022 — stoma / death	0 / 0	NR / NR	These outcomes could not be attributed confidently to the separable self-inserted subgroup and were therefore changed from inferred zeros to not reported/not separable.

#	Study / field	Original submission	Final revised value	Basis for correction
14	Fritz 2022 — stoma	1 (APR)	3	Primary-report re-verification showed that the original extraction captured only one operative category.
15	Maddah 2023 — stoma	NR	1	Primary-report re-verification.
16	Barone 1976 / Barone 1983 — cohort overlap	Possible overlap could not be excluded	No overlap	The study periods were verified as 1970–1974 and 1978–1981, respectively.

Abbreviations: APR, abdominoperineal resection; NR, not reported or not separable from the eligible subgroup; TAMIS, transanal minimally invasive surgery.

B. Corrections identified only after the review was expanded during revision

The following values were **not fields in the original 18-study submission table** or arose in studies added during the expanded review. They are therefore shown as corrections to an **earlier revision-stage extraction**, not as corrections to the original submission.

#	Study / field	Earlier revision-stage value	Final revised value	Basis for correction
1	Volpi 2012 — operative detail	Milking / colotomy not reported	Milking 0; colotomy 1; stoma 1	The primary report contains the operative detail; the earlier revision extraction recorded it as unavailable.
2	Brungardt 2021 — abdominal procedures	33	81	The value 33 represented a single procedure code within the authors' broader transperitoneal-operation category (n = 81).
3	Dahlberg 2019 — abdominal procedures	8	7, with one additional laparotomy flagged §	One laparotomy followed successful manual extraction and treated a perforation rather than retrieving the object.
4	Odagiri 2015 — abdominal procedures	168	98	The larger value double-counted overlapping categories taken from a secondary summary table.
5	Caliskan 2011 — abdominal procedures / deaths	10 / 2	11 / 3	The secondary summary table used during an intermediate extraction disagreed with the primary report.
6	Crass 1981 — N / abdominal procedures	29 / 14	18 / 4	The larger figures combined the retained-object cohort with a separate perforation cohort.
7	Rodríguez-Hermosa 2007 — milking / colotomy	0 / 0	NR / NR	The technique used in the two eligible laparotomies was not separately reported; zero had been an inference.

§ Abdominal operation was performed for perforation or another acute complication rather than primarily to extract the object.

C. Aggregate consequences of re-verification

Endpoint	Provenance of earlier value	Earlier value	Final synthesis
Included case series	Original submission	18	60
Abdominal procedures	Earlier revision-stage aggregate	413/2,677 across 56 series (15.4%)	428/2,867 across 59 series (14.9%)
Milking distinguished	Earlier revision-stage aggregate	57/173 across 41 series (32.9%)	64/181 across 42 series (35.4%)
Colotomy	Earlier revision-stage aggregate	85/244 across 40 series	85/254 across 42 series
Stoma	Earlier revision-stage aggregate	146 across 50 series	151 across 52 series
Mortality	Original-submission narrative claim	"Among modern series that reported mortality, no deaths were recorded."	54 reporting series; 49 reported zero deaths; 14 deaths in 5 series

Important provenance note. The original submission did **not** present a pooled or crude review-wide abdominal-procedure aggregate; it explicitly stated that abdominal-procedure rates were not pooled because cohort denominators differed. The review-wide values shown above for abdominal procedures, milking, colotomy, and stoma were calculated only during the expanded revision and are labelled accordingly.

D. Methodological changes made during revision

**Item**	**Original submission**	**Final revision**
Minimum series size	n ≥ 3	n ≥ 5
Age restriction	Adults	Cohorts containing minors were not excluded solely on the basis of age
Language	English-language/available-translation restriction	No language restriction; translation assistance used, with numerical values cross-checked against original-language tables
Databases	PubMed/MEDLINE and Embase; 818 records	PubMed/MEDLINE, Embase, Scopus, and Web of Science Core Collection; 6,018 records
Search structure	Foreign-body terms combined with a required operative/intervention clause	Operative AND-clause removed; controlled vocabulary added; intervention terms deliberately omitted from the retrieval filter
Additional search routes	No report-level other-methods pathway in the submitted PRISMA flow	Backward citation searching and targeted journal/archive searching added; 38 reports entered the other-methods pathway
Retrieval classification	Non-English records without an available translation were excluded at full-text screening; the submitted flow did not separately account for a not-retrieved pathway	Unavailable reports classified as not retrieved, not as exclusions; no abstract-only data used in synthesis
Registration	Not registered	Retrospective Open Science Framework registration, DOI 10.17605/OSF.IO/A2XES
JBI appraisal	Author-defined composite "low/moderate/high" categories	Item-level JBI judgments only; no composite score
Data verification	Values used as originally extracted	Every value carried from the original submission re-verified against the primary full text, with the stated exception that the eligible Ayantunde & Oke 2006 subgroup size (n = 16) was confirmed from a subsequent report by the same authors

E. Original-submission claims withdrawn or reframed after re-verification

**Claim in the original submission**	**Final disposition**
"Among modern series that reported mortality, no deaths were recorded."	Withdrawn. Deaths were identified in five included series; the final synthesis reports 14 deaths in five series, with 49 of 54 mortality-reporting series recording zero deaths.
"The algorithm in Figure 1 extends rather than repeats existing schemes."	Withdrawn/reframed. Stepwise escalation and intermediate techniques have precedents in the historical literature. The final manuscript presents the algorithm as a retrospectively characterised, hypothesis-generating synthesis rather than a novel or prospectively mandated pathway.

F. Final archival closure sweep before search freeze

During the final archival closure sweep, two additional eligible series were incorporated and the search was then frozen. **Trenin 2005** was included as a flagged non-separable mixed cohort (parent N = 143; abdominal procedures = 4; milking = 3). The whole-cohort denominator and abdominal count were retained from the report because the eligible inserted-object subgroup was not separable; classification of three abdominal procedures as delivery to the anus without bowel opening was supported by clarification from the corresponding author of a later Russian series, as disclosed in Supplementary Table S6. **Batmaz 2002** was included as an eight-patient Turkish series (abdominal procedures = 6; milking = 4; colotomy = 0; stoma = 3; death = 0). Two additional retrieved reports (Albayrak 2007 and an Akkaya textbook chapter) were excluded at full text, while Şahin 1995 and Ören 1991 were recorded as not retrieved. These additions account for the final PRISMA totals of **60 included series**, **67 full-text exclusions**, and **9 reports not retrieved**.

Notes on interpretation

1. Section A contains only values or claims that can be traced to the **submitted manuscript (ID 1933279)**.

2. Section B contains corrections made to values that appeared **only after the review was expanded during revision**.

3. “NR” is used when a value was not reported or could not be separated confidently from the eligible subgroup; inferred zeros were not retained.

4. The purpose of this table is transparency of revision, not to imply that every newly added study or variable was erroneous in the original submission.

Supplementary Table S4. Sensitivity analyses

S4.1 Excluding non-separable mixed cohorts

Mixed-cohort separability is a review-level eligibility variable and is independent of JBI item 1.

Study excluded	N in main synthesis	Abdominal procedures	Reason
Eftaiha 1977	30	3	eligible insertion-only subgroup not separable
Erverdi 1998	7	2	eligible insertion-only subgroup not separable
Ayantunde & Oke 2006	16	1	eligible insertion-only subgroup not separable
Coskun 2013	15	3	eligible insertion-only subgroup not separable
Ünver 2014	36	8	eligible insertion-only subgroup not separable
Cheung 2019	32	3	eligible insertion-only subgroup not separable
O'Farrell 2022	32	3	eligible insertion-only subgroup not separable
Odagiri 2015	648	98	eligible insertion-only subgroup not separable
Trenin 2005	143	4	eligible insertion-only subgroup not separable
Excluded subtotal	959	125	

Main synthesis: **428/2867 = 14.9%**.

After exclusion of the nine non-separable mixed cohorts: **303/1908 = 15.9%**.

Khan 2021 is retained because its eligible 15-patient subgroup is separable from the 22-patient parent cohort.

S4.2 Retaining series of three or four eligible patients

The minimum series size was raised from three to five eligible patients after screening had begun. Five reports excluded solely by that change contained an eligible per-anum retained foreign-body subgroup from which the operative route could be derived. This analysis retains them.

Study retained	Eligible N	Abdominal procedures	Note
Atila 2004	4	0	all four extracted transanally under anaesthesia
Pavlidis 2005	3	0	one of four reported patients had no retained object at operation and is not counted
Karateke 2013	3	0	one of four reported patients had removed the object himself before presentation and underwent repair of perforation; not counted
Sangar 2013	4	3	colotomy in three, of whom two received a stoma
Tofigh 2022	3	1	colotomy with primary closure; no stoma
Subtotal	17	4	

Main synthesis: **428/2867 = 14.9%**.

Retaining the five reports: **432/2884 = 15.0%**.

The proportion changed by **0.05 percentage points** and no direction of finding was reversed.

Supplementary Table S5. Full search strategies

All searches were run without restriction by language, publication date, or intervention. Coverage was from database inception to the date of the search. Records retrieved are reported as of the date of execution.

---

1. PubMed / MEDLINE

Interface https://pubmed.ncbi.nlm.nih.gov · **Date run** 5 August 2026 · **Records retrieved** 1,465

```
("Foreign Bodies"[Mesh] AND ("Rectum"[Mesh] OR "Rectal Diseases"[Mesh] OR
"Anal Canal"[Mesh] OR "Colon, Sigmoid"[Mesh])) OR "rectal foreign body"[tiab]
OR "rectal foreign bodies"[tiab] OR "colorectal foreign body"[tiab] OR
"colorectal foreign bodies"[tiab] OR "anorectal foreign body"[tiab] OR
"anorectal foreign bodies"[tiab] OR ((rectum[tiab] OR rectal[tiab] OR
anorectal[tiab] OR anorectum[tiab]) AND ("foreign body"[tiab] OR
"foreign bodies"[tiab]))
```

2. Embase

Interface https://www.embase.com (Advanced Search) · **Date run** 5 August 2026 · **Records retrieved** 2,312

```
'rectum foreign body'/exp OR ('foreign body'/exp AND ('rectum'/exp OR
'rectum disease'/exp OR 'anus'/exp OR 'sigmoid colon'/exp)) OR
'rectal foreign body':ti,ab OR 'rectal foreign bodies':ti,ab OR
'colorectal foreign body':ti,ab OR 'colorectal foreign bodies':ti,ab OR
'anorectal foreign body':ti,ab OR 'anorectal foreign bodies':ti,ab OR
((rectum:ti,ab OR rectal:ti,ab OR anorectal:ti,ab OR anorectum:ti,ab) AND
('foreign body':ti,ab OR 'foreign bodies':ti,ab))
```

3. Scopus

Interface https://www.scopus.com (Advanced document search) · **Date run** 5 August 2026 · **Records retrieved** 1,857

```
TITLE-ABS-KEY("rectal foreign body" OR "rectal foreign bodies" OR
"colorectal foreign body" OR "colorectal foreign bodies" OR
"anorectal foreign body" OR "anorectal foreign bodies") OR
```

(TITLE-ABS-KEY(rectum OR rectal OR anorectal OR anorectum OR "anal canal")

AND TITLE-ABS-KEY("foreign body" OR "foreign bodies"))

```

## 4. Web of Science Core Collection

**Interface** https://www.webofscience.com (Advanced Search, Query Builder) · **Editions** all · **Date run** 5 August 2026 · **Records retrieved** 384

```

TS=("rectal foreign body" OR "rectal foreign bodies" OR

"colorectal foreign body" OR "colorectal foreign bodies" OR

"anorectal foreign body" OR "anorectal foreign bodies") OR

(TS=(rectum OR rectal OR anorectal OR anorectum) AND

TS=("foreign body" OR "foreign bodies"))

```

---

## 5. Searching by other methods

### 5.1 Forward citation searching

Performed in Scopus on 6 August 2026 on the two prior systematic reviews of rectal or colorectal foreign bodies.

| Source | Citing records screened | Additional eligible studies |
|---|---|---|
| Kurer MA, et al. Colorectal Dis. 2010;12(9):851-61. | 88 | 0 |
| Ploner M, et al. Acta Gastroenterol Belg. 2020;83(1):61-5. | 17 | 0 |
| Total | 105 | 0 |

These 105 citing records are citation-level records; they are reported descriptively and are not included in the report-level count of 38 reports identified through other methods (Figure 2).

### 5.2 Backward citation searching

Reference lists were screened for all included series with 30 or more patients, all series published from 2010 onward, and six narrative or systematic reviews (Kurer 2010; Ploner 2020; Goldberg and Steele 2010; Cologne and Ault 2012; Tarasconi 2021 [WSES–AAST]; Pandey 2026).

### 5.3 Targeted journal and archive searching

Journals not indexed in the four databases and identified as likely to publish eligible reports were searched directly by volume, including Turkish, Russian, Japanese, Taiwanese, Tajik, Mexican, Thai, and Indian titles.

**Reports identified through other methods: 38** (included 10; not retrieved 5; excluded at full text 23; see Supplementary Table S2).

---
```

6. Record counts

	Records
PubMed / MEDLINE	1,465
Embase	2,312
Scopus	1,857
Web of Science Core Collection	384
Total from databases	6,018
Duplicates removed	−2,713
Records screened after de-duplication	3,305

Duplicates were removed by DOI and by normalised title.

---

7. Eligibility criteria

Population	Patients with a foreign body introduced per anum and retained in the rectum; cohorts containing minors were not excluded solely on the basis of age
Management	Any reported extraction or management pathway from which the transanal versus abdominal route could be determined
Comparator	Alternative operative approaches, where reported
Outcomes	Extraction route; milking versus opening the bowel; enterotomy, resection, or stoma; perforation; mortality; length of stay
Study design	Original case series of five or more eligible patients from which the operative route could be derived

Excluded — case reports and series of fewer than five eligible patients; reviews, guidelines, and textbooks without original patient data; ingested or orally introduced foreign bodies; iatrogenic and migrated devices; penetrating anorectal trauma without a retained object; mixed cohorts in which the eligible inserted-object subgroup was not separable and the prespecified retention criteria were not met; reports from which the extraction route or operative outcome could not be derived; duplicate or linked publications of an included cohort; conference abstracts.

Reports for which the full text could not be obtained are recorded as *not retrieved* in Figure 2 rather than as exclusions.

---

8. Notes

(1) Intervention terms were deliberately omitted from the search strings. An earlier version of this search combined foreign-body terms with a required operative-terms clause (surgery, laparotomy, extraction, and similar). That clause acted as a filter and excluded eligible series whose titles and abstracts did not contain those words. The strategy above searches the population only; eligibility by intervention and study design was assessed at screening.

(2) No language restriction was applied. Non-English reports were translated for screening and data extraction. Numerical outcomes were verified against the original tables, which are language-independent. Where an appraisal item could not be judged confidently from the translated text, it was rated *unclear*.

(3) Screening and appraisal. Screening was performed independently by two reviewers, with a third reviewer available to adjudicate disagreements. Critical appraisal used the JBI Critical Appraisal Checklist for Case Series and is reported at item level (Supplementary Table S7).

(4) Synthesis was a structured narrative synthesis; no meta-analysis was undertaken.

(5) Registration. The review was registered retrospectively on the Open Science Framework after data extraction had commenced (DOI 10.17605/OSF.IO/A2XES).

(6) Search freeze. After the final archival closure sweep documented in Supplementary Table S3, no further searching was undertaken. The final report-level other-methods pathway contains 38 reports.

Supplementary Table S6. Operative approach and outcomes in the 60 included case series

NR = not reported or not separable from the eligible subgroup. The present study is shown for comparison and is not included in the literature-review aggregates.

Symbols: † denominator is presentations/foreign bodies rather than unique patients; ‡ mixed parent cohort (eligible subgroup used where separable; otherwise the retained whole-cohort value is flagged); § abdominal operation was performed for perforation/another acute complication rather than primarily to extract the object; ¶ exact attribution could not be resolved from the primary report; # the data source did not permit the value to be derived; ‖ internal inconsistency in the primary report; ◆ linked publication exists; ※ extraction relied on available expanded primary-report material rather than a complete conventional full-text copy.

#	Study	Country	Period	N	Abdominal	Milking	Colotomy	Stoma	Death	Notes
1	Barone 1976 [51]	USA	1970–1974	23	0	0	0	0	0	‡
2	Eftaiha 1977 [7]	USA	1970–1975	30	3	1	2	1	0	†‡
3	Crass 1981 [75]	USA	1975–1980	18	4	2	2	0	0	‡‖
4	Barone 1983 [20]	USA	1978–1981	35	1	0	0	1	0	†‡§
5	French 1985 [52]	UK	1978–1983	9	1	0	0	1	0	
6	Nehme Kingsley 1985 [8]	USA	1976–1984	51	1	1	0	0	0	
7	Couch 1986 [9]	Australia	1968–1983	24	1	0	1	1	0	†‡‖
8	Yaman 1993 [78]	Canada	1975–1990	22	2	0	0	2	0	‡§
9	Cohen 1996 [53]	USA/UK	1983–1994	48	0	0	0	5	0	‡‖
10	Kouraklis 1997 [54]	Greece	1981–1994	21	4	0	1	3	0	
11	Ooi 1998 [10]	Singapore	1989–1997	17	2	2	0	1	0	‡
12	Erverdi 1998 [23]	Turkey	1992–1997	7	2	2	0	0	0	‡
13	Biriukov 2000 ※ [24]	Russia	1969–1998	112	5	5	0	1	0	
14	Subbotin 2000 ※ [55]	Russia	1975–1997	26	4	2	2	3	0	‖
15	Ruiz del Castillo 2001 [59]	Spain	1980–2000	17	7	NR	NR	5	0	
16	Batmaz 2002 [41]	Turkey	1998–2001	8	6	4	0	3	0	
17	Huang 2003 [22]	Taiwan	1979–2000	10	4	1	0	3	0	†§
18	Lake 2004 [11]	USA	1993–2002	87	8	1	5	2	0	†
19	Trenin 2005 [40]	Russia	5 yr ¶	143	4	3	0	2	NR	‡¶
20	Clarke 2005 [42]	South Africa	NR	13	5	1	3	2	0	¶
21	Ayantunde & Oke 2006 [43]	UK	2001–2004	16	1	NR	NR	0	0	‡
22	Rodríguez-Hermosa 2007 ◆ [47]	Spain	1997–2004	16	2	NR	NR	≤2	0	‡¶◆
23	Kim SD 2008 [56]	Russia	25 yr ¶	128	2	NR	NR	2	0	¶
24	Ono 2008 [58]	Japan	2001–2007	5	2	1	0	1	0	‖
25	Safioleas 2009 [26]	Greece	1981–2006	34	6	0	1	≥1	0	§¶
26	Caliskan 2011 [12]	Turkey	1988–2008	30	11	4	0	5	3	‡‖
27	Volpi 2012 [44]	Italy	2007–2010	10	2	0	1	1	0	
28	Buczyński 2012 [45]	Poland	2003–2011	8	1	0	1	1	0	

#	Study	Country	Period	N	Abdominal	Milking	Colotomy	Stoma	Death	Notes
29	Coskun 2013 [76]	Turkey	1999–2009	15	3	2	0	1	0	‡
30	Liu 2013 [32]	Taiwan	1990–2010	19	2	0	0	1–2	0	¶
31	Yildiz 2013 [14]	Turkey	1998–2013	25	2	NR	NR	NR	0	‡‖
32	Ünver 2014 [28]	Turkey	2006–2013	36	8	2	4	3	NR	‡
33	Daminova 2014 [50]	Tajikistan	2000–2013	54	12	NR	NR	≥1	1	‡‖¶◆
34	Odagiri 2015 [74]	Japan	2007–2013	648	98	NR	NR	35	8	†‡
35	Ayantunde 2016 [70]	UK	2008–2012	25	0	0	0	0	0	
36	Khubezov 2016 [27]	Russia	1999–2015	112	14	4	10	7	1	‡‖
37	Yılmaz & Cartı 2016 [57]	Turkey	NR	5	0	0	0	0	0	¶
38	Cawich 2017 [21]	Trinidad & Tobago	2009–2014	10	5	1	4	2	0	‖
39	Kokemohr 2017 [15]	Germany	2006–2016	20	2	0	2	0	0	†‖
40	Cinar 2018 [16]	Turkey	2006–2015	11	8	3	NR	5	0	‖
41	Cheung 2019 [30]	Taiwan	2007–2017	32	3	¶	¶	NR	NR	‡¶◆
42	Dahlberg 2019 [4]	Sweden	2009–2017	85	7	4	3	2	0	§¶
43	Heil 2020 [33]	Germany	2002–2017	33	3	0	2	0	0	‡‖
44	Kim JH 2020 [77]	Korea	2006–2018	14	7	2	4	1	0	
45	Bravo-Cuéllar 2021 [3]	Mexico	1999–2019	194	10	NR	NR	2	1	‖¶
46	Brungardt 2021 [71]	USA	2015–2018	109	81	NR	30	28	0	¶#
47	Khan 2021 [48]	Pakistan	2019	15	10	NR	NR	0	0	‡‖
48	Pang 2021 [31]	Malaysia	2018–2019	5	1	0	1	0	0	¶
49	Süleyman 2021 [46]	Turkey	2016–2020	12	2	1	0	1	0	
50	Fritz 2022 [96]	Germany	2006–2019	57	11	2	2	3	0	†‖
51	O'Farrell 2022 [49]	UK	2017–2021	32	3	1	0	1	0	‡◆
52	Zhang 2022 [82]	China	2012–2020	87	NR	NR	NR	NR	NR	‡#
53	Bisgin 2023 [94]	Turkey	2010–2020	21	7	3	0	4	0	‖◆
54	Frendt 2023 [79]	USA	2010–2020	78	12	NR	NR	NR	NR	#
55	Maddah 2023 [73]	Iran	2001–2019	14	1	0	1	1	0	‖
56	Egorkin 2024 [25]	Russia	2022–2024	47	1	1	0	0	0	‡
57	Ilkanich 2024 [18]	Russia	2019–2023	47	5	3	2	1	0	¶
58	Xu 2025 [72]	China	2019–2024	65	7	2	0	5	NR	‡‖
59	Mansour 2026 [19]	Israel	2014–2024	39	5	3	1	1	0	‖
60	Singh 2026 [83]	India	2021–2025	20	7	NR	NR	2	0	‖
—	Present study	Korea	2019–2023	22	8	2	2	3	0	comparison only*

Selected notes relevant to primary endpoints

- **Barone 1976:** retained-FB denominator = 23; five perforation cases in which the object had already passed before presentation were excluded from the retained-FB denominator.

- **Huang 2003:** 10 patients/12 presentations; four abdominal operations are recorded, but only one was undertaken primarily to retrieve the retained object; the other three were for perforation/sepsis management (§).
- **Rodríguez-Hermosa 2007:** parent cohort = 30 (16 anal introductions, 14 ingestions). Seven patients required laparotomy, five for perforation from ingested FBs and **two for anally introduced objects**; therefore Abdominal = 2 is attributable to the eligible subgroup. The operative extraction technique used in those two cases is not separately reported, so Milking and Colotomy are **NR**, not zero.
- **Trenin 2005:** the parent cohort of 143 includes 37 faecalomas and four ingested fish or bird bones; the four abdominal operations could not be attributed to the eligible inserted-object subgroup, so the whole-cohort denominator and abdominal count are shown (‡). Classification of three of the four abdominal procedures as delivery to the anus after laparotomy without opening the bowel lumen was supported by clarification from the corresponding author of a later Russian series. Study period is given as five years without stated calendar limits (¶).
- **Batmaz 2002:** two objects were removed transanally and six at laparotomy; in four of the six the object was milked to the anus without opening the bowel. Of the three stomas, two followed perforation and one followed division of the external sphincter during transanal delivery.
- **Khan 2021:** the eligible 15-patient subgroup is separable from the 22-patient parent cohort and remains in the main synthesis.
- **Zhang 2022:** the self-inserted subgroup (n=87) is separable, but final abdominal management after transanal failure is not reported for that subgroup; Abdominal = NR.
- **Present study:** abdominal approach = 8 (open 5, HALS 2, laparoscopic 1); milking without deliberate bowel incision = 2; deliberate extraction enterotomy with primary closure = 2; resection = 3; one additional abdominal case involved lavage and primary repair of a pre-existing contained perforation without diversion; stoma = 3. *Not included in review aggregates.*

Recalculated review aggregates

- Abdominal procedure: **428/2867 across 59 series = 14.9%**
- Milking distinction: **64/181 across 42 series = 35.4%**
- Colotomy: **85/254 across 42 series**
- Exact stoma count: **151 across 52 series**
- Mortality: **54 reporting series; 49 reported zero deaths**

Supplementary Table S7. Item-level JBI critical appraisal of the 60 included case series

Judgments are reported item by item; no composite score was calculated. Y = yes; N = no; U = unclear; NA = not applicable. "Not applicable" judgments are excluded from any denominator.

Study	1	2	3	4	5	6	7	8	9	10
Barone 1976	U	Y	Y	U	U	Y	Y	Y	Y	NA
Eftaiha 1977	U	Y	Y	U	U	Y	U	Y	Y	NA
Crass 1981	U	Y	Y	U	U	Y	U	Y	Y	NA
Barone 1983	Y	Y	Y	Y	N	Y	U	Y	Y	NA
French 1985	U	Y	Y	U	U	Y	Y	Y	Y	NA
Nehme Kingsley 1985	Y	Y	Y	U	U	Y	U	Y	Y	NA
Couch 1986	U	U	U	Y	N	Y	U	U	Y	NA
Yaman 1993	U	Y	Y	U	Y	Y	Y	Y	Y	NA
Cohen 1996	Y	Y	Y	Y	U	Y	Y	Y	Y	NA
Kouraklis 1997	Y	Y	Y	U	U	Y	U	Y	Y	NA
Ooi 1998	Y	Y	Y	Y	Y	Y	Y	Y	Y	Y
Erverdi 1998	U	Y	Y	U	U	Y	U	Y	Y	NA
Biriukov 2000 ※	Y	Y	Y	U	U	Y	U	U	Y	NA
Subbotin 2000 ※	Y	Y	Y	U	U	Y	U	U	Y	NA
Ruiz del Castillo 2001	U	Y	Y	U	U	Y	U	Y	Y	NA
Batmaz 2002	U	Y	Y	U	U	Y	Y	Y	Y	NA
Huang 2003	Y	Y	Y	U	U	Y	Y	Y	Y	NA
Lake 2004	Y	Y	Y	Y	U	Y	Y	Y	Y	Y
Clarke 2005	U	Y	Y	U	U	Y	U	Y	Y	NA
Trenin 2005	U	Y	Y	U	U	Y	Y	U	Y	NA
Ayantunde & Oke 2006	Y	Y	Y	U	U	Y	U	Y	Y	NA
Rodríguez-Hermosa 2007	Y	Y	Y	Y	Y	Y	Y	Y	Y	Y
Kim SD 2008	U	Y	Y	U	U	Y	U	Y	Y	NA
Ono 2008	Y	Y	Y	U	U	Y	Y	Y	Y	NA
Safioleas 2009	U	Y	Y	Y	U	Y	U	U	Y	NA
Caliskan 2011	U	Y	Y	U	U	Y	Y	Y	Y	NA
Volpi 2012	Y	Y	Y	U	N	Y	Y	U	Y	NA
Buczyński 2012	Y	Y	Y	U	U	Y	Y	Y	Y	NA
Coskun 2013	U	Y	Y	U	U	Y	Y	Y	Y	NA
Liu 2013	Y	U	U	Y	U	Y	N	U	Y	NA
Yildiz 2013	Y	Y	Y	U	U	Y	Y	Y	Y	NA
Ünver 2014	Y	Y	Y	Y	Y	Y	Y	Y	Y	NA
Daminova 2014	U	Y	Y	U	U	Y	N	U	U	NA
Odagiri 2015	Y	U	U	Y	Y	Y	N	U	Y	Y
Ayantunde 2016	Y	Y	Y	Y	U	Y	Y	Y	Y	NA
Khubezov 2016	U	Y	Y	U	U	Y	U	Y	Y	NA

Study	1	2	3	4	5	6	7	8	9	10
Yılmaz & Cartı 2016	U	Y	Y	U	U	Y	Y	Y	Y	NA
Cawich 2017	U	Y	Y	Y	N	Y	Y	U	Y	NA
Kokemohr 2017	Y	Y	Y	Y	Y	Y	U	Y	Y	NA
Cinar 2018	Y	Y	Y	U	U	Y	U	Y	U	NA
Cheung 2019	U	Y	Y	Y	U	Y	N	U	Y	NA
Dahlberg 2019	Y	Y	Y	Y	Y	Y	Y	Y	Y	Y
Heil 2020	Y	Y	Y	Y	N	Y	U	Y	Y	NA
Kim JH 2020	Y	Y	Y	U	U	Y	Y	Y	Y	NA
Bravo-Cuéllar 2021	U	Y	Y	Y	N	Y	Y	U	Y	NA
Brungardt 2021	Y	Y	Y	Y	U	Y	N	Y	N	Y
Khan 2021	Y	U	U	Y	Y	Y	U	U	U	NA
Pang 2021	U	Y	Y	U	U	Y	Y	Y	U	NA
Süleyman 2021	U	U	Y	U	N	Y	U	Y	Y	NA
Fritz 2022	Y	Y	Y	Y	Y	Y	Y	Y	Y	U
O'Farrell 2022	Y	U	U	U	U	Y	U	U	Y	NA
Zhang 2022	Y	Y	Y	Y	Y	Y	Y	U	Y	N
Bisgin 2023	Y	Y	Y	Y	Y	Y	Y	Y	Y	NA
Frendt 2023	Y	Y	Y	Y	U	Y	N	Y	Y	U
Maddah 2023	Y	Y	Y	U	U	Y	Y	Y	Y	NA
Egorkin 2024	Y	Y	Y	U	U	Y	Y	Y	Y	NA
Ilkanich 2024	Y	Y	Y	U	U	Y	Y	Y	Y	NA
Xu 2025	Y	U	U	U	U	Y	U	U	Y	Y
Mansour 2026	U	Y	Y	Y	N	Y	N	U	Y	Y
Singh 2026	Y	U	U	U	U	Y	N	N	Y	NA

Checklist items (JBI Critical Appraisal Checklist for Case Series; Munn Z, et al. JBI Evid Synth. 2020;18(10):2127-33):

1, Were there clear criteria for inclusion in the case series? 2, Was the condition measured in a standard, reliable way for all participants? 3, Were valid methods used for identification of the condition for all participants? 4, Did the case series have consecutive inclusion of participants? 5, Did the case series have complete inclusion of participants? 6, Was there clear reporting of the demographics of the participants? 7, Was there clear reporting of clinical information of the participants? 8, Were the outcomes or follow-up results of cases clearly reported? 9, Was there clear reporting of the presenting sites'/clinics' demographic information? 10, Was statistical analysis appropriate?

Item 1 was judged solely on whether the original report stated clear criteria for inclusion in its own case series. Separability of an inserted-foreign-body subgroup under the eligibility criteria of this review was recorded separately as a review-level variable (Supplementary Table S6 footnote ‡; Supplementary Table S4) and did not determine the item-1 judgment.

Item 10 was judged "not applicable" only where the report did not undertake a statistical analysis whose appropriateness could be appraised; otherwise the reported analysis was judged yes, no, or unclear. One nationwide administrative-database study reported between-group significance testing and logistic modelling and was therefore appraised rather than classed as not applicable.

※ Extraction and appraisal for these two reports relied on available expanded primary-report material rather than a complete conventional full-text copy; the values were checked against the original report.

Summary of final judgments by item (60 studies)

Item	Yes	No	Unclear	Not applicable
1	36	0	24	0
2	52	0	8	0
3	53	0	7	0
4	24	0	36	0
5	11	8	41	0
6	60	0	0	0
7	30	8	22	0
8	42	1	17	0
9	55	1	4	0
10	8	1	2	49

Supplementary Figures

Supplementary Figure S1. Friable porcelain cup that fragmented during attempted transanal extraction. (A) Coronal and (B) axial computed tomography showing the object within the rectum. (C) The retrieved fragments. Transanal extraction was abandoned after the cup fractured; the fragments were removed at laparotomy through an extraction enterotomy closed primarily, without resection or diversion. Friability, rather than size, determined the operative route.

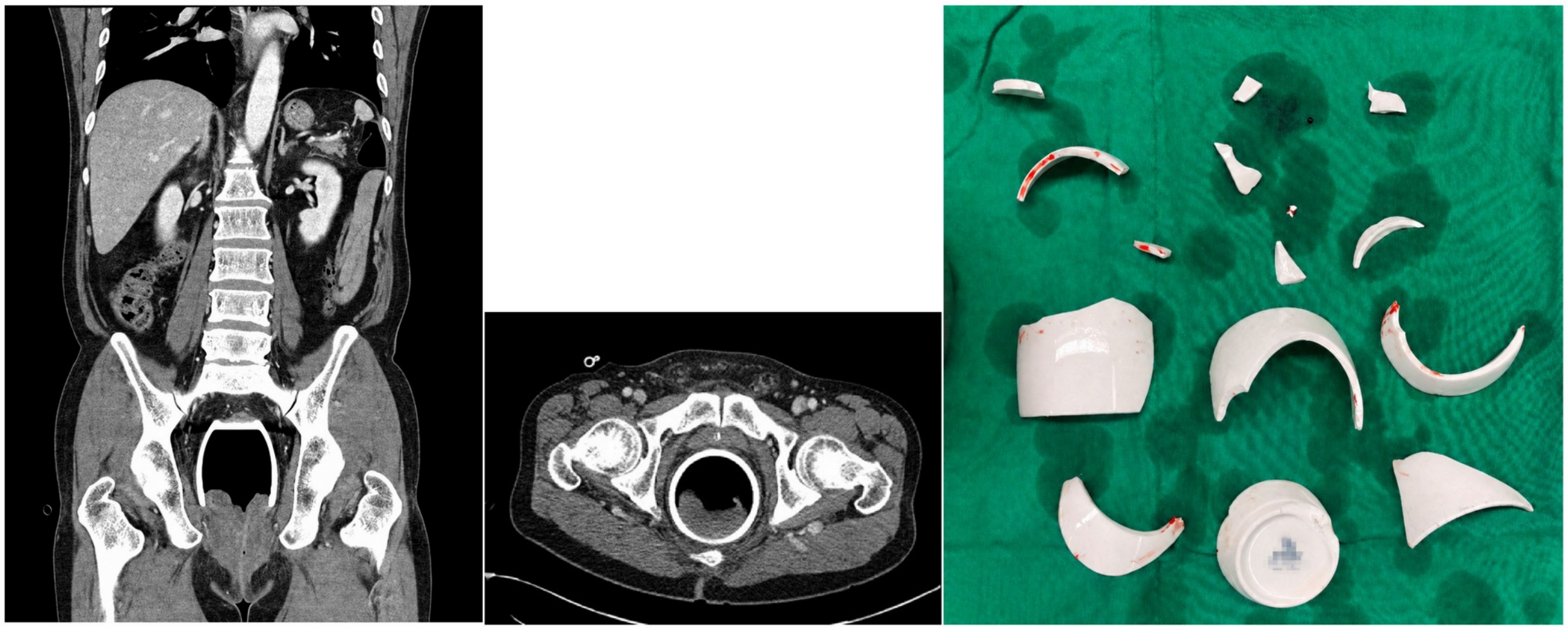

(A) **(B)** **(C)**

Supplementary Figure S2. Rigid wooden umbrella handle spanning the rectosigmoid junction, with its proximal end above it. (A) Coronal and (B) axial computed tomography at the level of the rectosigmoid junction. (C) The retrieved object. After transanal extraction failed, the object was milked to the anus at hand-assisted laparoscopy and delivered transanally; the bowel was not opened and no stoma was formed.

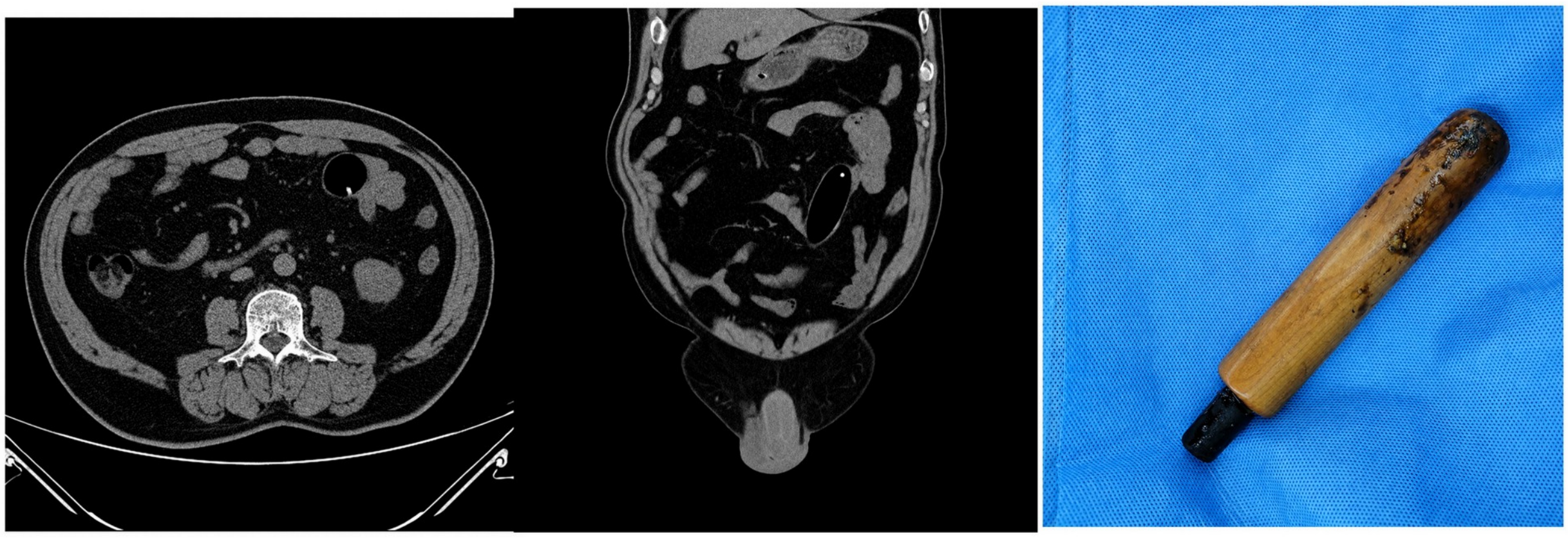

(A) (B) (C)

Supplementary Figure S3. Dome-shaped shampoo cap with no graspable edge. (A) Plain abdominal radiograph. (B) The retrieved object. Repeated transanal attempts failed because the smooth convex surface offered no purchase for instruments; the object was removed at hand-assisted laparoscopy through an extraction enterotomy closed primarily. Shape, rather than dimension, prevented transanal delivery.

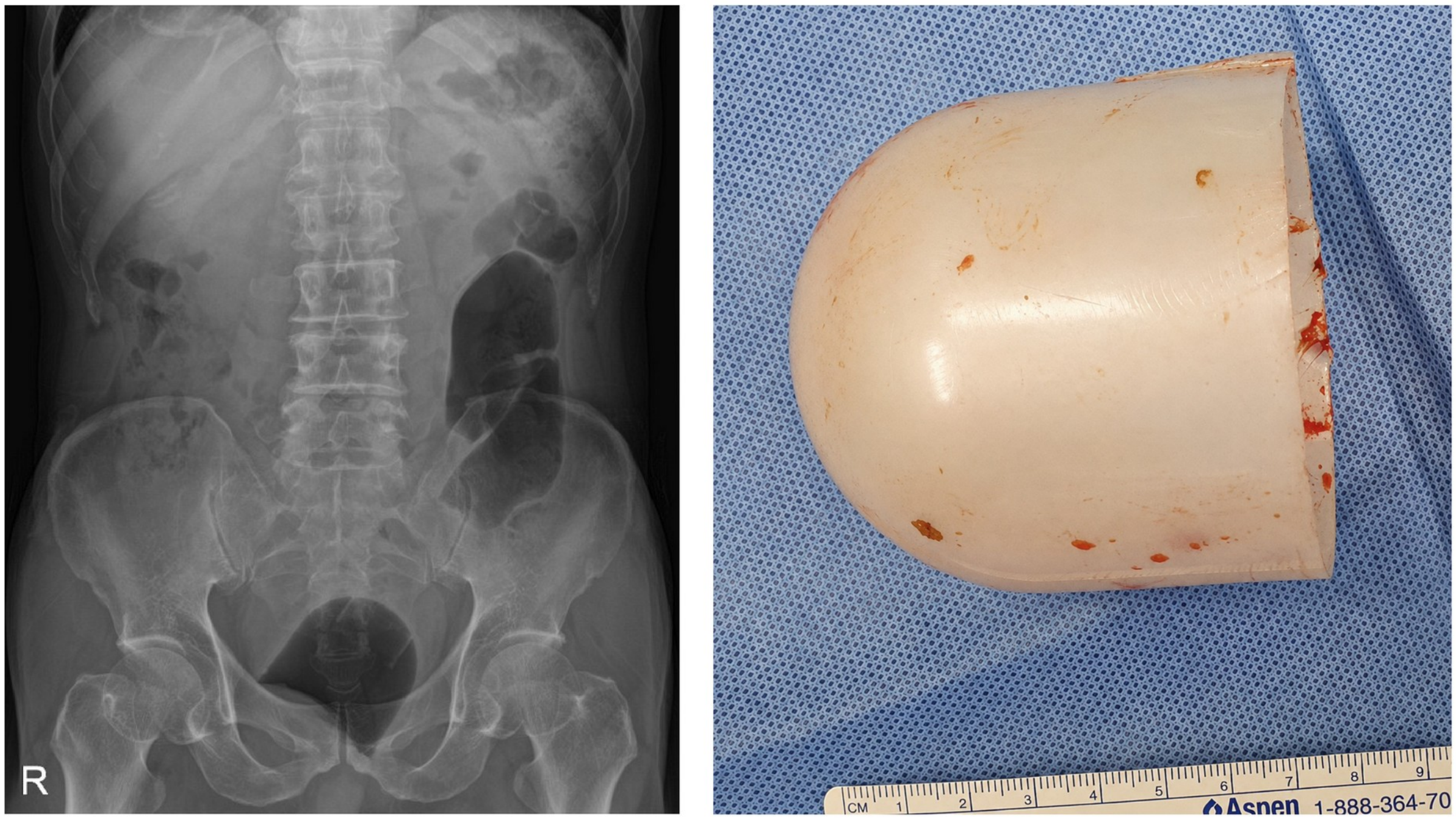

(A) (B)
